# Social Performance and Firm Risk: Impact of the Financial Crisis

**DOI:** 10.1007/s10551-016-3017-x

**Published:** 2016-02-11

**Authors:** Kais Bouslah, Lawrence Kryzanowski, Bouchra M’Zali

**Affiliations:** 1 Centre for Responsible Banking & Finance, School of Management, University of St Andrews, Scotland, UK; 2 Senior Concordia University Research Chair in Finance, Concordia University, Montreal, Canada; 3 AICRI, CRSDD-ESG-UQAM, Université du Québec à Montréal, Montreal, Canada

**Keywords:** Financial crisis, Volatility, Idiosyncratic risk, Social performance, Strengths, Concerns, G32, M14

## Abstract

This paper examines the impact of the recent financial crisis (2008–2009) on the relation between a firm’s risk and social performance (SP) using a sample of non-financial U.S. firms covering the period 1991–2012. We find that the relation between SP and risk is significantly different in the crisis period (post-crisis period) compared to the pre-crisis period. SP reduces volatility during the financial crisis. The risk reduction potential of SP is mainly due to the strengths component of SP. Since the relation of risk is stronger with SP strengths than SP concerns, this implies an asymmetric relation between these SP components and a firm’s risk. Specifically, strengths act as a risk reduction tool during an adverse economic environment.

## Introduction

During the last two decades, the concept of corporate social responsibility (CSR) grew in importance within the financial community. Several indicators support this claim. First, there has been an emergence and growth of specialized investment firms whose role is to monitor the behavior of firms in social domains and to provide social ratings for these firms (e.g., MSCI ESG STATS^1^). Second, several mutual funds and indices that select firms on the basis of CSR criteria have emerged (e.g., Dow Jones Sustainability Indexes, Domini 400 Social Index, Calvert Social Index). Third, there is an increased interest among investors in CSR issues. As of 2010, assets engaged in socially responsible investing (SRI) represent 12.2 % of all assets under management in the U.S. and 19.1 % in Canada (SIO 2010; SIF 2010). Also, major institutional investors from different countries have signed the Principles for Responsible Investment (PRI) protocol launched in April 2006.^2^ Finally, most firms, especially larger ones, produce specific reports or dedicate a specific section of their annual reports to discuss CSR issues.

This growing importance of CSR has fueled much research which examines the link between CSR or social performance (SP) and a firm’s attributes from different perspectives.^3^ Unfortunately, the numerous empirical studies of the relationship between SP and various measures of profitability, cost of capital, shareholder value/wealth, financial performance (including measures of risk), and the stock price performance yield mixed and inconclusive results (Pava and Krausz 1996; Margolis and Walsh 2003; Orlitzky et al. 2003; Starks 2009; Lee and Faff 2009; Baron et al. 2011; Oikonomou et al. 2012). This may be attributable to measurement problems associated with SP and omitted variables not included in the models used (Ullmann 1985). This may also be attributable to the differences in the conceptualisation of how SP affects a firm’s attributes. For example, it is still not clear whether and how SP affects a firm’s risk.^4^

Generally, previous studies hypothesize that SP will affect either systematic or idiosyncratic risk.^5^ Studies examining the impact of SP on systematic risk (market beta) argue that idiosyncratic risk is irrelevant since it can be diversified away through diversification. For example, Salama et al. (2011) find a negative relationship between a SP measure combining two dimensions (Community and Environment) and systematic risk for a sample of U.K. firms for the 1994–2006 period. Oikonomou et al. (2012) find a negative (positive) relation between an aggregate strengths (concerns) measure based on KLD data and systematic risk for S&P 500 firms (including utilities and financial firms) for the 1992–2009 period.

Although portfolio theory suggests that only systematic risk is relevant for asset pricing in perfect markets, a considerable body of empirical evidence suggests that idiosyncratic risk is also priced in financial markets (e.g., Goyal and Santa-Clara 2003; Ang et al. 2006; Fu 2009).^6^ Thus, some studies focus on the impact of SP on idiosyncratic risk. For example, Boutin-Dufresne and Savaria (2004) find a negative relationship between idiosyncratic risk and an aggregate measure of SP that combines several SP dimensions for a sample of Canadian firms over the period 1995–1999. Lee and Faff (2009) find that portfolios of firms having lower SP (proxied by the inclusion in the Dow Jones Sustainability Index) outperform portfolios of firms with superior SP. They conclude that higher returns for lagging SP firms compensate for the relatively higher idiosyncratic risk associated with such firms. Goss (2012) finds that aggregate measures of concerns (strengths) based on a principal components analysis of KLD data are positively (negatively) related to idiosyncratic risk measured using a vector autoregressive model, and that the relation is stronger for concerns than strengths. This asymmetrical financial effect is also found in other studies (e.g., Kappou and Oikonomou 2014; Jiraporn et al. 2014).

The objective of this paper is to examine the impact of the recent financial crisis (2008–2009) on the relation between a firm’s risk and social performance (SP) using a sample of non-financial U.S. firms covering the period 1991–2012. In particular, we examine this relationship during and after the financial crisis. This is highly relevant for several reasons. First, the financial crisis provides a natural setting to test main theories advanced in the CSR literature regarding the link between Risk and SP, e.g., the risk mitigation view and the overinvestment view. The risk mitigation view (a risk management argument based on the stakeholder theory) suggests a negative relationship between SP and firm risk because higher SP may decrease the likelihood of negative events at the firm level, and allow the firm to be better prepared for difficult periods (e.g., financial crises, economic recessions, compliance with more stringent future regulations). In contrast, the overinvestment view (an agency theory argument) suggests a positive relationship between SP and firm risk because of managerial entrenchment. During the financial crisis, almost all firms, including healthier ones, experienced increased volatility. If the risk mitigation view holds, the increased volatility of firms with high SP should be lower relative to firms with low SP during this period. The reverse should be observed if the overinvestment view holds.

Second, we argue in this paper that SP influences a firm’s total risk (stock return volatility) through its impact on idiosyncratic risk, and that this effect will be stronger during the financial crisis and potentially in the post-crisis period. In particular, SP affects idiosyncratic risk through its impacts on relationship-based intangible assets (e.g., trust, brand, reputation, employee moral, and customer loyalty). The potential cash flows from these intangible assets depend on the firm’s relationships with its stakeholders and the related assessments these stakeholders make regarding the firm’s activities (Godfrey 2005). These relationship-based intangible assets are expected to be more valuable during the financial crisis period. The examination of the post-crisis period allows us to examine whether this valuation persists over time or reverts back to its pre-crisis level.

Our major findings can be summarized as follows. First, the relation between SP and risk varies over time (is dynamic) and depends on market conditions. Our results indicate that the relation between SP and risk is significantly different in the crisis period (post-crisis period) compared to the pre-crisis period. Second, a one standard deviation increase in the aggregated social performance or SP (strengths minus concerns) index for a firm reduces its volatility by about 1.18–1.84 % during the financial crisis. The risk reduction potential of SP during the financial crisis is mainly due to the strengths component of SP. A one standard deviation increase in the aggregate measure of strengths (Str) decreases a firm’s volatility (idiosyncratic risk) by about 0.83–2.57 % (0.58–2.43 %) during the financial crisis. There is also some evidence suggesting that a one standard deviation increase in the aggregate measure of concerns (Con) increases total risk by 2.34 % during the financial crisis. Third, the relation between strengths and risk is stronger than the relation between concerns and risk during the financial crisis. This implies an asymmetric relation between the social performance components and a firm’s risk with strengths acting as a risk reduction tool during adverse economic conditions (e.g., financial crises, economic recessions).

The results of our study contribute to the literature in several ways. First, our study assesses the risk effects associated with SP during and after the financial crisis. We provide a direct test of the risk management hypothesis where SP can be used strategically by firms to control risk which is consistent with a large literature showing that firms hedge to reduce cash flows volatility and the costs of financial distress (e.g., Stulz 2002). Because of market imperfections, risk management matters and can be priced in financial markets (Stulz 2002; Sharfman and Fernando 2008). Second, unlike previous studies, we document an asymmetric effect of SP on a firm’s risk during and after the financial crisis where Strengths reduce volatility and idiosyncratic risk, and Concerns have no or little impact on risk. Thus, simply avoiding concerns did not help manage firm risk during the financial crisis. Firms need to have SP strengths in order to enjoy risk reduction during a crisis period. Finally, by investigating the impact of SP on a firm’s risk during and after the recent financial crisis, we provide strong empirical evidence suggesting that SP (in particular Strengths) is a risk reduction tool in difficult periods such as a severe financial or economic crisis. This fact was a common belief, but had not been tested directly in the previous empirical CSR literature.

The remainder of the paper is organized as follows. “Theoretical Framework and Research Hypotheses” section formulates the theoretical framework and research hypotheses. “Data and Sample Selection” section describes the data and sample selection procedure. “Methodology” section describes the methodology used to test our hypotheses. “Empirical Results” and “Robustness Checks” sections present and analyze our empirical results. “Conclusion” section concludes.

## Theoretical Framework and Research Hypotheses

### Effect of Social Performance on Risk

The CSR literature suggests three theoretical arguments that could explain how SP influences a firm’s risk: the risk mitigation view, theoretical models relating SP to expected returns, and the overinvestment view. The first two arguments predict a negative relation between SP and risk, while the overinvestment argument predicts a positive relationship.

The risk mitigation view (a risk management argument based on the stakeholder theory) predicts that SP is negatively related to firm risk. More specifically, this argument suggests that CSR investments (i.e., higher SP) can generate moral capital or goodwill among stakeholders, which provide insurance-like protection that reduces a firm’s risk exposure, i.e., preserves rather than generates financial performance (Godfrey 2005; Godfrey et al. 2009).^7^ This moral capital creates relational wealth in different forms among different stakeholder groups, e.g., affective commitment among employees, legitimacy among communities and regulators, trust among suppliers and partners, credibility and enhanced brand among customers, and higher attractiveness for investors (Godfrey 2005). The key point is that this moral capital has value as it disposes stakeholders to hold beliefs about the firm which influence their behaviors towards the firm (Luo and Bhattacharya 2009). Thus, “*CSR based moral capital creates value if it helps stakeholders attribute the negative event to managerial maladroitness rather than malevolence, and temper their reactions accordingly*” (Godfrey et al. 2009, p. 428).

Firms with higher SP will have higher moral capital which translates into a more favorable evaluation of the firm in the eyes of various stakeholder groups (e.g., consumers, employees and investors). In particular, higher moral capital provides insurance-like protection for the firm’s shareholder wealth by creating goodwill and mitigating negative stakeholders’ assessments when they are adversely affected in the event of a crisis. For example, a firm’s relationships with its key stakeholders will be tempered by the higher moral capital accumulated (e.g., customers’ loyalty and investors’ trust will suffer to a lesser extent). In other words, stakeholders will impose less severe sanctions on the firm with higher moral capital in the case of negative events. Also, higher SP helps to decouple the negative shocks (events) from the rest of the organization (Bansal and Clelland 2004), protect its public image, relieve regulatory pressure, and insulate the firm from scrutiny (Luo and Bhattacharya 2009). Consistent with this argument, Godfrey et al. (2009) find, using an event study methodology, that a SP measure combining the strengths of two dimensions (community and diversity) is positively related to the two-day cumulative abnormal returns following negative legal/regulatory actions against firms.

Furthermore, higher (lower) SP may reduce (increase) financial and operating risks (McGuire et al. 1988), and/or risk associated with social issues (Feldman et al. 1997; Sharfman and Fernando 2008; El Ghoul et al. 2011). Sharfman and Fernando (2008) argue that risk management of social or environmental issues is theoretically synonymous with strategic risk management. For instance, CSR investments (e.g., emissions and pollution reduction) reduce a firm’s risk from known and unknown hazards, and consequently reduce the number of potential claimants on a firm’s cash flows (e.g., potential fines, compensation, settlements, compliance costs, clean-up costs in the case of environmental accidents, or problems associated with poor working conditions). Firms with lower social performance may face several risks (e.g., damage to brand image and reputation, lower favorable investor recognition).

The risk mitigation view suggests that higher SP through CSR investments generates moral capital which creates relational wealth in different forms among different stakeholders (i.e., market-based intangible assets). This relational wealth reduces uncertainty about a firm’s future cash flows and, therefore reduces a firm’s risk. This is achieved through the insurance-like protection for a firm’s idiosyncratic intangible assets provided by CSR investments.

Theoretical models of the relationship between SP and expected returns (e.g., Heinkel et al. 2001; Mackey et al. 2007; Fama and French 2007) also suggest that SP is negatively related to firm risk. These models assume differences in investor preferences that can lead to segmented capital market pricing based on SP. Specifically, traditional investors make investment decisions based solely on financial criteria (anticipated payoffs and the access to overall consumption they provide) while socially responsible investors make investment decisions based on both financial and non-financial criteria (e.g., SP). Unlike traditional investors, socially responsible investors get additional utility from holding stocks chosen based on their SP because they have tastes for such assets as consumption goods that are unrelated to returns (Fama and French 2007). The main prediction of these models is the existence of price differences induced by demand differences for different types of stocks. Socially responsible stocks will have an excess demand which leads to lower risk and expected return (overvalued stocks). In contrast, socially irresponsible stocks (i.e., stocks having lower SP) will have a weak demand due to the “neglect effect,” which leads to higher risk and expected returns to compensate for lower risk sharing opportunities (undervalued stocks).

The main prediction derived from the theoretical models of the relationship between SP and expected returns is similar to that derived from the equilibrium model with incomplete information developed by Merton (1987) and imperfect markets due to market or regulatory frictions developed by Mao (1971), Levy (1978), and Kryzanowski and To (1982) where different investors hold different portfolios of risky assets in equilibrium. This leads to a differential “neglect effect” for stocks and segmented markets (or price differences induced by demand differences for different types of stocks).^8^ In particular, the model of Merton (1987) predicts that a firm’s risk is negatively related to the size of that firm’s investor base (i.e., the number of its shareholders). In turn, this suggests a negative relationship between SP and firm risk since a higher SP is expected to increase the investor base. Lee and Faff (2009) argue that the model of Merton (1987) is consistent with the argument that the risk management and transparency practices associated with SP are valued by investors.

In contrast to the aforementioned arguments, the overinvestment view (a managerial opportunism argument based on agency theory) suggests a positive relationship between SP and firm risk because of managerial entrenchment. For example, managers may choose to improve their firm’s SP score at the expense of shareholders by over-investing in CSR activities in order to build their own personal reputations as good social citizens (Barnea and Rubin 2010) or to generate support from social and environmental activists in order to reduce the probability of their replacement in a future period (Cespa and Cestone 2007). Surroca and Tribó (2008) provide empirical evidence suggesting that a firm’s SP may form part of a manager’s entrenchment strategy. They also find that the combination of entrenchment strategies and higher SP have negative effects on financial performance.

### Effect of Social Performance on Risk During and After the Financial Crisis

CSR expenditures can be expected to be lower given poorer economic conditions or when firms are more financially constrained. Ullman (1985) argues that economic demands will have priority over social demands in periods of low profitability. Branca et al. (2012) show theoretically and empirically that firms invest less in CSR activities (e.g., donate less) when the business cycle is unfavorable (e.g., the recent financial crisis), independently of the market structure. It seems reasonable to expect the current state of the economy (macroeconomic context or conditions) to have an effect on the financial performance of firms as well as on their SP. Under adverse macroeconomic conditions (e.g., negative shocks to demand), firms must decide whether to restrict their CSR expenditures in order to save resources, or use CSR to differentiate themselves more effectively (Branca et al. 2012). This is a legitimate argument. However, in this paper we look at this problem from a different perspective. Instead of asking the question of whether firms tend to reduce, maintain, or increase CSR expenditures because of adverse macroeconomic conditions, we ask the following question: can SP be a risk reduction tool in periods of adverse macroeconomic conditions?

Examining the relation between a firm’s risk and its SP during and after the financial crisis is relevant for two main reasons. First, the financial crisis provides a natural setting to test the main theories advanced in the CSR literature regarding the link between Risk and SP, e.g., the risk mitigation view and the overinvestment view. During the financial crisis, almost all firms experienced increased volatility. If the risk mitigation view holds, the increased volatility of firms with high SP should be lower relative to firms with lower SP. The reverse should be observed if the overinvestment view holds. Second, we argue in this paper that SP influences a firm’s total risk (stock return volatility) through its impact on idiosyncratic risk,^9^ and that this effect will be stronger during the financial crisis and potentially in the post-crisis period. In particular, SP affects idiosyncratic risk through its impacts on relationship-based intangible assets (e.g., trust, brand, reputation, employee moral, and customer loyalty). The potential cash flows from these intangible assets depend on the firm’s relationships with its stakeholders and the related assessments these stakeholders make regarding the firm’s activities (Godfrey 2005). Those relationship-based assets (i.e., relational wealth) are intangible and idiosyncratic to the firm (Godfrey 2005). In other words, relational wealth is heterogeneous between firms and idiosyncratic to specific firm-stakeholder relationships. These relationship-based intangible assets are expected to be more valuable during the financial crisis period. The examination of the post-crisis period allows us to examine whether this valuation persists over time or reverts back to its pre-crisis level.

It is well known that there are points in time (e.g., credit crises, economic recessions) when investors perceive firms to be more risky than during other times. Regardless of the economic prospects for firms, the markets suddenly become “more risky” leading to lower stock prices (i.e., lower firm values). During periods of economic recession, the average volatility increases, but the volatility increase of more risky firms (e.g., those with lower SP) could be dramatic. In a flight to safety during economic recessions or slowdowns, many investors seek the haven of less risky securities (e.g., U.S. Treasury bonds). More socially responsible firms (those with higher SP) could also be considered as less risky, and thus more safe relative to similar firms with lower SP. Investors may consider firms with higher (lower) SP as being less (more) risky investments because they may link SP with a higher quality of management (McGuire et al. 1988; Waddock and Graves 1997). Lee and Faff (2009) argue that firms with higher SP are able to reduce their idiosyncratic (business) risk relative to firms with lower SP. Based on the risk mitigation view as well as theoretical models relating SP to expected returns, we develop the following hypothesis stated in its alternative form:

#### **H**_**1**_^**A**^

SP is negatively related to firm risk, in particular idiosyncratic risk, during the financial crisis.

SP is an aggregate measure of the social performance of a firm at a given point in time. More specifically, it represents the difference between strengths (positive CSR) and concerns (negative CSR). It is important to distinguish explicitly between the strengths and concerns because they are conceptually distinct constructs (Mattingly and Berman 2006). They are likely to have different effects on firm risk since these two SP components are not strongly correlated (0.24 in our data). Furthermore, the possibility of substitution or compensation effects (e.g., Greenwashing) exists. Firms may undertake CSR investments to increase their strengths in order to compensate for current or future concerns.

The hypothesized negative relation between SP and risk could come either from strengths, concerns or both. For example, Mishra and Modi (2013) find that the idiosyncratic risk is negatively related to strengths, and positively related to concerns. In this case, the negative relation between SP and risk comes from both strengths and concerns. However, Oikonomou et al. (2012) find that systematic risk is positively and strongly related to concerns. In this case, the negative relation between SP and risk comes mainly from concerns. Lankoski (2009) argues that the economic impacts of SP are more positive for issues that reduce negative externalities (e.g., reducing or avoiding concerns) than for issues that generate positive externalities (e.g., having or increasing strengths). Several previous studies document the economic impact of concerns (e.g., Frooman 1997; Godfrey et al. 2009; Oikonomou et al. 2012; Goss and Roberts 2011; Goss 2012). Concerns are expected to have a positive impact on a firm’s risk because they are more likely to affect both traditional and socially responsible investors. It is reasonable to expect both types of investors to punish firms for concerns, i.e., socially irresponsible behavior such as product recalls, oil spills, sweatshop operations, or employee hazards in the workplace. It is also reasonable to expect such a market penalty to happen during normal market conditions as well as in period of economic crisis or recession. The penalty could even be higher in the latter case.

In this paper, we extend this literature by examining the less studied case where the negative relation between SP and risk comes mainly from strengths. In particular, we argue that SP reduces volatility and idiosyncratic risk during the financial crisis, and that the risk reduction potential of SP is mainly due to the strengths component of SP. Although investors may not agree on the value of the strengths and on their impacts (Ioannou and Serafeim 2014; Edmans, 2011), we hypothesize that this could not be the case during the financial crisis. For example, the risk mitigation view suggests that firms with higher SP (i.e., those having strengths) will develop goodwill or moral capital that functions as “insurance-like” protection during bad times. In other words, strengths could be very useful during adverse macroeconomic conditions where investors seek less risky securities (a flight to safety).^10^ We believe that in adverse macroeconomic conditions, the role of non-financial attributes such as SP becomes very important. In this case, SP will play the role of a simple scale that enables investors to readily assess a firm’s risk similar to credit ratings which provide information about default likelihood and the financial health of firms. Jiraporn et al. (2014) find that more socially responsible firms enjoy more favorable credit ratings. That is, firms with higher strengths will be perceived as being less risky firms. This is highly valued by investors during adverse macroeconomic conditions. In short, SP (in particular Strengths) could be a risk reduction tool in difficult periods such as a severe financial or economic crisis. Therefore, we expect an asymmetric effect where the relation with firm risk is stronger for the strengths measure than for the concerns measure during a crisis. This leads to our second hypothesis stated in its alternative form:

#### **H**_**2**_^**A**^

There is an asymmetric effect where the relation with firm risk is stronger for the strengths measure than for the concerns measure of SP during the financial crisis.

## Data and Sample Selection

The social performance data for U.S. firms from the MSCI ESG STATS (formerly KLD Research & Analytics, Inc or KLD) database have been used extensively by other researchers (e.g., Mattingly and Berman 2006; Kempf and Osthoff 2008; Gregory and Whittaker 2013). Based on calendar year-end data, the database provides *Strength* ratings and *Concern* ratings for several binary indicators (i.e., 1 or zero value for presence or absence) for seven qualitative dimensions and only *Concern* ratings for several indicators of six exclusionary dimensions. It also provides total counts of all strengths and concerns in each of these 13 dimensions. The seven **qualitative dimensions** are Community, Diversity, Employee Relations, Environment, Product, Human Rights (formerly “*non*-*US operations*” before 2002), and Corporate Governance (formerly “*Other”* category before 2002). The six **exclusionary dimensions** are Alcohol, Gambling, Firearms, Military, Nuclear Power, and Tobacco. Since the exclusionary dimensions are fundamentally different from the qualitative dimensions, we follow previous research (see e.g., Harjoto and Jo 2008; Jiraporn et al. 2014) and do not include them in our analysis. The KLD rating (either strength or concern) for a particular indicator within a particular qualitative dimension is a binary variable which is equal to one if the firm has a strength or concern, and zero otherwise (i.e., has no strength or concern). For example, a firm that implements pollution prevention and recycling programs will have a positive score along the environmental dimension. Conversely, a firm that has poor union relations and retirement benefits concerns will have a negative score along the employee relations dimension.^11^ KLD data are free of survivorship bias (Kempf and Osthoff 2008) and does not change much from year to year (Gregory and Whittaker 2013). KLD data are the most recognized and accepted in the literature (Jiraporn et al. 2014).

Our final sample consists of an unbalanced panel dataset of 28,110 firm-year observations for all non-financial and non-utility firms covered by MSCI ESG STATS or KLD and three other databases (CRSP, COMPUSTAT and I/B/E/S) over the period 1991–2012 based on each firm’s CUSIP. For many firms, we perform a hand-check to ensure a successful merging process. We obtain stock prices, stock returns, trading volumes, and shares outstandings from CRSP. Accounting data are obtained from COMPUSTAT. Analyst earnings forecasts data are obtained from Thomson Reuters I/B/E/S.

## Methodology

### Measuring Social Performance

Most empirical studies using KLD data combine the various SP dimensions into one aggregate SP measure using different methods (e.g., Graves and Waddock 1994; Waddock and Graves 1997; Harjoto and Jo 2008; Jiraporn et al. 2014). For example, Harjoto and Jo (2008) net the average concerns and strengths for each of the five KLD dimensions considered, and then compute an arithmetic average index of SP. In this paper, we follow Harjoto and Jo (2008) and use strengths ${\text{Str}}_{it}$, concerns ${\text{Con}}_{it}$ and their aggregation ${\text{SP}}_{it} = {\text{Str}}_{it} - {\text{Con}}_{it}$, where the former two are respectively given by^12^:

1 $${\text{Str}}_{it} = \frac{1}{D}\sum\limits_{d = 1}^{D} {\left[ {\frac{1}{{N_{\text{STR}} }}\sum\limits_{l = 1}^{L} {{\text{Strength}}_{l} } } \right]_{it} }$$

2 $${\text{Con}}_{it} = \frac{1}{D}\sum\limits_{d = 1}^{D} {\left[ {\frac{1}{{N_{\text{CON}} }}\sum\limits_{j = 1}^{J} {{\text{concern}}_{j} } } \right]}_{it}$$

where *d* refers to the KLD dimension, and *D* is the total number of KLD dimensions for a given year *t* and firm *i*. $N_{\text{STR}}$ and $N_{\text{CON}}$ are total maximum possible numbers of strengths and concerns, respectively, for a given KLD dimension for a given year.^13^

### Measuring Firm Risk

We measure a firm’s total risk by the annualized standard deviation from the daily stock returns over the past year. We compute systematic risk (market beta) and idiosyncratic (unsystematic) risk using the basic CAPM and the four-factor Carhart (1997) model, respectively, using the factors obtained from Kenneth French’s web site.^14^ The latter model is given by:

3 $$R_{it} - R_{ft} = \alpha_{i} + \beta_{iM} (R_{Mt} - R_{ft} ) + \beta_{is} {\text{SMB}}_{t} + \beta_{ih} {\text{HML}}_{t} + \beta_{iu} {\text{UMD}}_{t} + \varepsilon_{it}$$

where

*R*_*it*_ is the return of firm *i* for day *t*. *R*_*ft*_ is the risk-free rate (daily Treasury-bill rate). $(R_{Mt} - R_{ft} )$ is the excess return on the market portfolio (CSRP value-weighted index) for day *t*. SMB_*t*_ is the difference between the returns on portfolios of “small” and “big” capitalization stocks for day *t*. HML_*t*_ is the difference between the returns on portfolios of “high” and “low” book-to-market stocks for day *t*. UMD_*t*_ is the difference between the daily returns on portfolios of “high” and “low” prior return (months −12 to −2) stocks. *ε*_*it*_ is the stochastic error term, assumed to be IID normal with mean zero and constant variance or idiosyncratic risk $\sigma_{{\varepsilon_{i} }}^{2}$.^15^ The CAPM model is obtained by dropping the last three factors. All models are estimated using factor returns. Systematic risk (market beta) and unsystematic (idiosyncratic) risk are estimated for both models using the previous year’s daily excess returns for each firm-year observation. Idiosyncratic (unsystematic) risk is measured as the annualized standard deviation of the residuals from these estimations. This follows common practice in the literature where risk is measured using higher frequency (daily) data over shorter time periods of up to 1 year to better capture its time-varying nature.^16^

### Relation Between Firm Risk and Social Performance

Our objective is to examine the impact of the recent financial crisis on the link between social performance and a firm’s risk. To do so, we estimate the following regressions:

4 $${\text{Risk}}_{it} = \alpha_{0} + \alpha_{1} {\text{SP}}_{it} + \alpha_{2} {\text{SP}}_{it} \times {\text{Crisis}}_{it} + \alpha_{3} {\text{SP}}_{it} \times {\text{Postcrisis}}_{it} + \delta X_{it} + \varepsilon_{it}$$

5 $${\text{Risk}}_{it} = \alpha_{0} + \alpha_{11} {\text{Str}}_{it} + \alpha_{12} {\text{Str}}_{it} \times {\text{Crisis}}_{it} + \alpha_{13} {\text{Str}}_{it} \times {\text{PostCrisis}}_{it} + \alpha_{21} {\text{Con}}_{it} + \alpha_{22} {\text{Con}}_{it} \times {\text{Crisis}}_{it} + \alpha_{23} {\text{Con}}_{it} \times {\text{PostCrisis}}_{it} + \delta X_{it} + \varepsilon_{it}$$

where Risk_*it*_ and SP_*it*_ are the risk and the social performance measure for firm *i* at time *t*, respectively. *X*_*it*_ is a vector of firm-specific characteristics, industry factors, and economic or market-wide factors that affect a firm’s risk. *δ* is a vector of the related regression coefficients.

The “pre-crisis” period is 1991–2007, the “crisis” period is 2008–2009, and the “post-crisis” period is 2010–2012. As such, the variable “Crisis” (“PostCrisis”) is a dummy variable that takes a value of 1 for years 2008 and 2009 (years 2010, 2011, and 2012) and zero for the other years in the sample period.^17^ The interactions variables ${\text{SP}}_{it} \times {\text{Crisis}}_{it}$ and ${\text{SP}}_{it} \times {\text{PostCrisis}}_{it}$ are our main variables of interest. Using Eq. (4) to illustrate, the coefficient $\alpha_{1}$ gives the estimated effect of SP on the measure of risk for the pre-crisis period and the base value for the crisis and post-crisis periods. The coefficient $\alpha_{2}$ gives the additional effect of SP on the measure of risk for the crisis period, whereas the coefficient $\alpha_{3}$ gives the additional effect of SP on the measure of risk for the post-crisis period. If $\alpha_{2}$ is significant, it indicates that the relationship between risk and SP is different between the two periods (pre-crisis and crisis periods). If $\alpha_{3}$ is significant, it indicates that the relationship between risk and SP is different between the two periods (pre-crisis and post-crisis periods). The total effect of SP on the measure of risk for the crisis period is given by the sum $(\alpha_{1} + \alpha_{2} )$, whereas the total effect of SP on the measure of risk for the post-crisis period is given by the sum $(\alpha_{1} + \alpha_{3} )$. Thus, $\alpha_{1}$ informs us about the sign and significance of the relation between risk and SP in the pre-crisis period, whereas $(\alpha_{1} + \alpha_{2} )$ and $(\alpha_{1} + \alpha_{3} )$ inform us about the sign and significance of this relation in the crisis and post-crisis periods, respectively. Similarly, in Eq. (5), the coefficient *α*_12_ represents the additional impact of Strengths on risk during the financial crisis relative to the pre-crisis period, whereas *α*_13_ does the same for the post-crisis period. The coefficients *α*_22_ and *α*_23_ represent the additional impact of Concerns on risk during the financial crisis and the post-crisis period relative to the pre-crisis period.

Table 1 lists the determinants of firm risk used herein. Firm-specific characteristics (expected sign) include Firm size (−),^18^ Book-to-Market (B/M) ratio (+),^19^ Financial leverage (+),^20^ Expected return (+),^21^ Stock liquidity (−),^22^ Cash flow risk (+), Investment-to-asset ratio (−),^23^ Expected growth in earnings (+), Default risk (+), and the size of the investor base (−).^24^

**Table 1:** Definition of the variables

Variable	Measure
Aggregate social performance (SP)	Aggregate (composite) measure of social performance, which combines strengths and concerns (SP = Str−Con)
Strengths (Str)	Aggregate measure of strengths
Concerns (Con)	Aggregate measure of concerns
Systematic risk (betadcapmw)	The market beta derived from the CAPM using the previous year’s daily excess returns for each firm-year observation
Systematic risk (betad4ffw)	The market beta derived from the four-factor Carhart (1997) model using the previous year’s daily excess returns for each firm-year observation
Idiosyncratic risk (IVcapmdw)	The annualized standard deviation of the residuals derived from the CAPM model estimated using the previous year’s daily excess returns
Idiosyncratic risk (IV4ffdw)	The annualized standard deviation of the residuals derived from the four-factor Carhart (1997) model estimated using the previous year’s daily excess returns
Firm’s total risk (voldw)	The annualized standard deviation from the daily stock returns over the past year

Firm size (lnmkteq)	The natural logarithm of the market value of common equity at the most recent fiscal year end to account for the highly skewed nature of this variable
Book-to-Market (B/M) ratio (bmw)	The ratio of the book-to-market value of common equity as of the most recent fiscal year end
Financial leverage (netlevw)	We follow Bates et al. (2009) by using a net leverage measure calculated as the ratio of long-term debt minus cash & marketable securities to the market value of common equity using values for the most recent fiscal year end
Expected return (rmedinfw)	The expected return is proxied by the implied cost of equity capital (rmedinfw) calculated using the implied cost of capital (ICC) methodology (see the Appendix). We also consider the annualized return from the previous year’s daily stock returns (ret1y). The ICC is a much less noisier measure of expected returns than realized returns. Therefore, we use rmedinfw in our regressions as proxy for expected returns
Stock liquidity (both level and risk)	The level of liquidity (avgturnover) is proxied by the average daily share turnover (daily shares traded divided by daily shares outstanding), and the liquidity risk is proxied by the coefficient of variation (cvturnover) of this measure over the previous year. The Amihud illiquidity measure (illiq) is computed as in Amihud (2002)
Cash flow risk	Dispersion of analyst forecasts: the cross-sectional standard deviation of either one-year-ahead earnings forecasts (dispeps1w) or long-term growth in earnings forecasts (displtg). We expect a positive relation between the dispersion of analyst forecasts and firm risk because a higher dispersion in earnings forecasts implies greater disagreement between analysts about forecasted earnings Standard deviation of the return on assets (ROA): the standard deviation of ROA (sdroa5yw) is computed over the five previous years up to the fiscal year end date of each firm-year observation
Investment-to-asset ratio (Investmentw)	We use three proxies for investment: capital expenditures divided by total assets (capex), R&D expenditures divided by total assets (rd), and advertising expenses divided by total assets (ad). Investment-to-asset ratio is the sum of these three variables divided by total assets
Expected growth in earnings (expgrthw)	The mean annualized five-year earnings growth rate from I/B/E/S (where available, otherwise estimated as the implicit growth in forecasted earnings from year 1 to year 2)
Default risk	Altman’s (1993) Zscore: $Z_{\text{score}} = 1.2 \times \left( {\frac{\text{NWC}}{\text{TA}}} \right) + 1.4 \times \left( {\frac{\text{RE}}{\text{TA}}} \right) + 3.3 \times \left( {\frac{\text{EBIT}}{\text{TA}}} \right) + \left( {\frac{\text{Sales}}{\text{TA}}} \right) + 0.6 \times \left( {\frac{\text{MVEquity}}{\text{BVTL}}} \right)$ where NWC is net working capital (current assets—current liabilities), RE is retained earnings, EBIT is earnings before interest and taxes, MVEquity is the market value of total equity (common and preferred stocks), BVTL is total liabilities (current and long-term liabilities), and TA is total assets. A higher value of the Zscore indicates a lower likelihood of default
Size of investor base (inv_basew)	The number of common ordinary shareholders divided by common shares outstanding

We estimate Eqs. 4 and 5 using three estimation methods: two-way cluster regression model, two-way fixed effects panel data regression model, and instrumental variables (IV) regression model. Any robust effect should hold regardless of the methodology used. The two-way cluster model is the robust approach of Petersen (2009) which clusters standard errors on both firm and time effects. We include industry dummy variables, based on the Fama and French (1997) industry classification, to control for industry fixed effects. We also include year dummy variables to control for the effects of changing economic conditions on a firm’s risk. For the two-way fixed effects panel data regression model, we control for firm fixed effects as well as time effects. The fixed effects approach mitigates the omitted variable bias by controlling for unobservable firm characteristics that remain constant through time. We do not include industry dummies in the fixed effects model as those effects are already subsumed in the firm fixed effects.

#### Correcting for the Endogeneity of Social Performance^25^

The regression specification in Eqs. (4 or 5) assumes that social performance SP is exogenous. However, SP may be endogenous because some of the regressors (e.g., firm size and industry) and unobserved variables that are omitted in the model could affect both SP and the firm’s risk. In such cases, the explanatory variable *SP*_*it*_ is likely to be endogenous. This endogeneity problem could produce a spurious relationship.

To correct for this potential endogeneity problem, we use the instrumental variables (IV) regression method estimated using the two-step efficient generalized method of moments (GMM)^26^:

6 $${\text{SP}}_{it} = \gamma + \eta Z_{it} + \theta Y_{it} + \omega_{it}$$

7 $${\text{Risk}}_{it} = \alpha_{0} + \alpha_{1} {\text{SP}}_{it}^{*} + \alpha_{2} {\text{SP}}_{it}^{*} \times {\text{Crisis}}_{it} + \alpha_{3} {\text{SP}}_{it}^{*} \times {\text{PostCrisis}}_{it} + \delta X_{it} + \varepsilon_{it}$$

where *Z*_*it*_ denotes instruments, and *Y*_*it*_ denotes variables that affect social performance (e.g., firm size and industry). Chosen instruments should be correlated with SP but have zero or low correlation with the disturbance in the structural model for the firm’s risk (Eq. 7). We follow Jiraporn et al. (2014) and use the average SP of neighboring firms (geographically proximate firms) as well as the average industry SP as instruments. Jiraporn et al. (2014) show that SP is significantly influenced by the SP of the surrounding firms in the same three-digit zip code, an effect possibly due to investor clientele, local competition, and/or social interactions. The variation in SP across zip codes is likely exogenous because it is not correlated with corporate financial policies or outcomes. The U.S. Postal Service allocates zip codes exclusively based on efficiency in postal delivery, not corporate financial policies or outcomes (Jiraporn et al. 2014).

We also use the average industry SP as an instrument. Firm risk may be related to firm-level SP, but it is less likely related to industry level SP. Thus, the changes in SP at the industry level are more likely to be exogenous. Moreover, the use of the average industry SP allow us to control for industry differences in the SP scores because social issues are different for different industries and are time-varying (Carroll 1999). Each industry has different configurations of stakeholders with disparate degrees of activism on the issues (Carroll 1999). To construct the two instruments, we follow the same methodology as Jiraporn et al. (2014). We conduct a statistical test (Hansen J statistic) of overidentifying restrictions to ensure the validity of the used instruments. If the Hansen J statistic (overidentification test of all instruments) is not statistically significant (i.e., *p* value higher or equal to 0.1), our instrumental variables are valid. In the first stage regression, we use our instruments in order to predict ${\text{SP}}_{it}$, ${\text{Str}}_{it}$, or ${\text{Con}}_{it}$. In the second stage regression, we use the fitted values ($SP_{it}^{*}$, ${\text{Str}}_{it}^{*}$, or ${\text{Con}}_{it}^{*}$) obtained in the first stage [Eq. (6)] as the explanatory variables instead of their original values, and run the regression in Eq. (7). We include industry and year dummy variables in both stages and we only report the results of the second stage estimation.

#### Cross-Sectional Determinants of SP

In Eq. (6), *Y*_*it*_ is a vector of firm-specific characteristics, industry factors, and market-wide factors that could affect SP. *θ* is the related vector of coefficients. Previous empirical studies find that SP can be affected by several firm characteristics which include risk (e.g., beta and standard deviation of returns), firm size, leverage ratio, book-to-market ratio, capital expenditures, R&D expenditures, advertising expenses, and industry.^27^ Moreover, recent studies find that SP is negatively related to the cost of equity capital (Feldman et al. 1997; Sharfman and Fernando 2008; El Ghoul et al. 2011), and financial distress or default risk (Goss, 2007).

Based on theoretical arguments and the empirical evidence reported in these previous studies, the firm-specific characteristics considered in the SP model used herein are firm size (lnmkteq), Book-to-Market ratio (bmw), net leverage (netlevw), the cost of equity capital (ICC), the level of stock liquidity (avgturnover), the liquidity risk (cvturnover), dispersion of analyst forecasts (dispeps1w), investment-to-asset ratio (investment), expected growth in earnings (expgrthw), default risk (zscorew), and investor base (inv_basew).^28^

The variable investor base (inv_basew) is included to control for ownership structure following the empirical evidence reported in previous studies showing a significant relationship between SP and some measures of ownership structure such as institutional and insiders’ ownership (e.g., Brammer and Pavelin 2006; Mahoney and Roberts 2007; Barnea and Rubin 2010; Harjoto and Jo 2011). We expect this variable to be positively related to SP based on theoretical arguments (e.g., Heinkel et al. 2001; Mackey et al. 2007). In all our regressions, standard errors are adjusted for both heteroskedasticity and clustering of observations.

## Empirical Results

### Univariate Analysis^29^

Table 2 presents the sample distribution for all firms (except financial and utility firms) covered by KLD between 1991 and 2012. In order to facilitate the interpretation of our results, we need to characterize our sample clearly. To do this, we begin by dividing the sample into four groups based on Strengths (Str) and Concerns (Con) measures as defined in Table 1. The “toptier” and “lowtier” group includes all firms having only Strengths and only Concerns, respectively. The “medtier” group includes all firms having both Strengths and Concerns. The “zerotier” group includes all firms having neither Strengths nor Concerns. Table 2 shows that 11 % of our sample firms have only Strengths, 35 % have only Concerns, 44 % have both Strengths and Concerns, and 10 % have neither Strengths nor Concerns. The “toptier” group has decreased during and after the financial crisis (13 % before the crisis and 8 % after the crisis), whereas the “lowtier” group has increased (from 30 % pre-crisis to 49 % post-crisis). The “medtier” group increased from 46 % pre-crisis to 50 % during the crisis, and decreased to 32 % post-crisis. The “zerotier” group decreased during the crisis, but returned to its pre-crisis level after the crisis. The “medtier” group is the dominant group before and during the crisis (46–50 %), whereas the “lowtier” group is the dominant group after the crisis (49 %).

**Table 2:** Sample distribution by year and social performance scores

Year	Toptier	Lowtier	Medtier	Zerotier	Total
1991	153	100	141	105	499
1992	145	118	179	60	502
1993	107	124	232	35	498
1994	79	109	280	21	489
1995	87	102	284	22	495
1996	107	83	266	43	499
1997	76	98	300	25	499
1998	87	93	294	26	500
1999	91	90	305	18	504
2000	80	80	327	21	508
2001	129	142	378	170	819
2002	104	170	392	126	792
2003	306	613	681	517	2117
2004	258	794	936	236	2224
2005	222	831	920	178	2151
2006	152	795	1080	114	2141
2007	158	798	1056	126	2138
2008	172	793	1082	114	2161
2009	174	773	1084	164	2195
2010	108	1267	701	138	2214
2011	39	1351	718	3	2111
2012	353	500	593	608	2054
Total	3187	9824	12,229	2870	28,110
1991–2012	0.11	0.35	0.44	0.10	1
1991–2007	0.13	0.30	0.46	0.11	1
2008–2009	0.08	0.36	0.50	0.06	1
2010–2012	0.08	0.49	0.32	0.12	1

This table presents the sample distribution for all firms (except financial and utility firms) covered by KLD between 1991 and 2012. The sample is divided into four groups based on Strengths (Str) and Concerns (Con) measures as defined in Table 1. The toptier group includes all firms having positive Str, but zero Con. The lowtier group includes all firms having positive Con, but zero Str. The medtier group includes all firms having both positive Str and positive Con. The zerotier group includes all firms having zero Str and zero Con

Table 3 presents the descriptive statistics of the social performance measures (panel A), the risk measures (Panel B), and the explanatory variables (Panel C) for all firms (except financial and utility firms) covered by KLD between 1991 and 2012. Panel A of Table 3 shows that the mean (median) values of the aggregate measures of social performance SP which combine strengths and concerns are negative (−0.03) suggesting that concerns are, on average, higher than strengths. This observation is confirmed when the combined measure is split into two aggregate measures of strengths and concerns. The mean (median) values of concerns are 0.07 (0.05), whereas the mean (median) values of strengths are 0.04 (0.02).

**Table 3:** Descriptive statistics of the KLD scores, the risk measures, and the explanatory variables for the period 1991–2012

	Mean	Median	Standard deviation	Min	Max	Skewness	Kurtosis	N
Panel A: SP measures								
SP	−0.03	−0.03	0.09	−0.464	0.749	1.341	10.354	28,110
Str	0.04	0.02	0.08	0	0.843	3.836	23.756	28,110
Con	0.07	0.05	0.07	0	0.681	1.828	8.974	28,110
Panel B: risk measures								
voldw	0.459	0.408	0.225	0.142	1.528	1.654	6.863	27,450
IVcapmdw	0.395	0.349	0.201	0.118	1.436	1.846	8.108	27,450
IV4ffdw	0.381	0.336	0.195	0.113	1.403	1.887	8.354	27,450
betadcapmw	1.205	1.151	0.507	0.131	2.679	0.528	3.151	27,450
betad4ffw	1.090	1.064	0.407	0.030	2.265	0.337	3.361	27,450
Panel C: independent variables								
lnmkteq	7.226	7.079	1.611	−3.090	13.348	0.430	3.220	27,945
bmw	0.494	0.407	0.421	0	4.652	3.477	26.500	27,944
Leveragew	0.453	0.156	1.274	0	20.235	9.589	124.806	27,875
netlevw	0.289	0.059	1.155	−1.669	17.474	8.796	109.749	27,996
ret1yw	0.146	0.152	0.443	−1.295	1.576	−0.051	4.650	27,134
rmedinfw	0.095	0.090	0.045	0.009	0.312	1.662	8.460	25,254
avgturdw	2.464	1.897	2.006	0.189	10.754	1.820	6.863	27,450
cvturdw	0.053	0.045	0.026	0.022	0.189	2.156	9.262	27,450
dispeps1w	0.092	0.040	0.145	0	0.990	3.820	20.289	24,966
rd	0.046	0.004	0.117	0	7.791	17.785	860.451	27,996
ad	0.015	0	0.043	0	0.963	6.417	66.826	27,996
capex	0.059	0.039	0.087	−0.519	9.235	44.197	4496.643	27,959
investmentw	0.119	0.088	0.117	−0.006	1.265	3.231	20.464	27,996
expgrthw	0.168	0.140	0.151	0	1	3.517	18.560	25,863
zscorew	4.702	3.408	6.571	−107.123	60.896	2.251	35.499	28,011
displtg	0.046	0.032	0.056	0	1.642	8.505	151.963	18,055
sdroa5yw	0.061	0.033	0.083	0.004	0.537	3.442	17.048	28,058
inv_basew	0.137	0.041	0.251	0.000	2.458	4.355	30.348	27,535

This table presents the descriptive statistics of the social performance measures (panel A), the risk measures (Panel B), and the explanatory or control variables (Panel C) for all firms (except financial and utility firms) covered by KLD between 1991 and 2012. Except for the social performance measures and dummy variables, the variables are winsorized (*w*) at the 1st and 99th percentiles. The variables are as defined in Table 1

Based on Panel B of Table 3, the mean (median) total risk is 0.45 (0.40) using one-year daily returns. The mean (median) idiosyncratic risk is 0.39 (0.34) using the CAPM and quite similar at 0.38 (0.33) using the four-factor model. The mean (median) systematic risk (market beta) is 1.20 (1.15) using the CAPM and lower at 1.09 (1.06) using the four-factor model.^30^ Panel C of Table 3 reports descriptive statistics for our explanatory variables.

Table 4 reports the means and t-test results (*p* values) for the difference in means for risk and social performance measures across two periods: pre-crisis (1991–2007) and post-crisis (2010–2012). Our objective is to investigate whether the post-crisis risks and social performances are higher or lower than their levels in the pre-crisis period. For the whole sample, the mean total risk of 44 % for the post-crisis period is significantly higher than its level of 40 % in the pre-crisis period. The systematic risk (market beta) has slightly decreased after the crisis to 1.07 relative to its pre-crisis level of 1.09. However, the idiosyncratic risk reverted back to its pre-crisis level in the post-crisis period of 35 %. Although both the mean strengths and concerns have increased significantly after the crisis, the aggregate SP improved slightly (−0.02 in the post-crisis period relative to −0.03 in the pre-crisis period).

**Table 4:** Risk and Social Performance before and after the financial crisis

	Toptier	Lowtier	Medtier	Zerotier	All
voldw					
Pre-crisis	0.40	0.41	0.38	0.43	0.40
Post-crisis	0.38	0.50	0.38	0.43	0.44
*p* value (difference)	0.002	0.000	0.168	0.909	0.000
IV4ffdw					
Pre-crisis	0.36	0.36	0.33	0.38	0.35
Post-crisis	0.30	0.39	0.28	0.37	0.35
*p* value (difference)	0.000	0.000	0.000	0.368	0.501
betad4ffw					
Pre-crisis	1.04	1.12	1.08	1.10	1.09
Post-crisis	1.12	1.05	1.08	1.08	1.07
*p* value (difference)	0.000	0.000	0.843	0.103	0.001

	Toptier	Lowtier	Medtier	Zerotier	All
SP					
Pre-crisis	0.05	−0.07	−0.02	0	−0.03
Post-crisis	0.16	−0.10	0.06	0	−0.02
*p* value (difference)	0.000	0.000	0.000	nd	0.007
Str					
Pre-crisis	0.05	0	0.06	0	0.04
Post-crisis	0.16	0	0.17	0	0.07
*p* value (difference)	0.000	nd	0.000	nd	0.000
Con					
Pre-crisis	0	0.07	0.09	0	0.06
Post-crisis	0	0.10	0.12	0	0.09
*p* value (difference)	nd	0.000	0.000	nd	0.000

This table presents the means and *t* test results (*p* values) for the difference in means for risk and social performance measures across two periods. Years 1991 through 2007 are defined as Pre-crisis period, and Years 2010 through 2012 are defined as Post-crisis period. The variables reported are: total risk (voldw), idiosyncratic risk (IV4ffdw), systematic risk (beta4ffdw), net social performance (SP), Strengths (Str), and Concerns (Con). All variables are defined in Table 1. The sample is divided into four groups based on Strengths (Str) and Concerns (Con) measures. The toptier group includes all firms having positive Str, but zero Con. The lowtier group includes all firms having positive Con, but zero Str. The medtier group includes all firms having both positive Str and positive Con. The zerotier group includes all firms having zero Str and zero Con. The acronym “nd” means “not defined”

The total risk and the idiosyncratic risk of the “toptier” group have decreased, whereas this group’s systematic risk has increased in the post-crisis period. In contrast, both total and idiosyncratic risks have increased and the systematic risk has decreased in the post-crisis period for the “lowtier” group. After the crisis, the SP of the “toptier” group increased significantly and that of the “lowtier” group decreased significantly. The total and systematic risks of the “medtier” group reverted back to their pre-crisis levels in the post-crisis period. However, the idiosyncratic risk of the “medtier” group has decreased significantly from its pre-crisis level. The SP of the “medtier” group has improved significantly in the post-crisis period, although both the mean strengths and concerns have increased significantly after the crisis. The improved SP for the “medtier” group stems from the fact that the increase in its strengths is higher than the increase in its concerns. In summary, Table 4 indicates that risk and social performance have changed during and after the financial crisis. Total risk is higher post-crisis compared to its pre-crisis level. Both the mean strengths and concerns increased significantly after the crisis, although the overall SP has slightly improved relative to its pre-crisis level.

In untabulated results, we find that the aggregate measure of social performance (SP), which combines strengths and concerns, has a positive correlation of 0.68 with the strengths (Str) and a negative correlation of −0.54 with concerns (Con). The correlations between strengths and concerns is positive but relatively low (0.24), which supports the notion that they are different concepts and should be treated separately in empirical work. We also find (results not reported here) that all risk measures are negatively correlated with all SP measures (SP, Str and Con). However, the magnitude of the negative correlation between risk and Str is higher than that between risk and Con.

Table 5 reports the correlation coefficients among the independent variables. Only a few of the explanatory variables are highly correlated as expected. For example, the correlation coefficient between investment and R&D is 0.68. Except for these special cases, the correlation coefficients are relatively low overall, which mitigate any multicollinearity concerns that could affect the regression results.

**Table 5:** Correlation coefficients among the explanatory variables

		(1)	(2)	(3)	(4)	(5)	(6)	(7)	(8)	(9)
lnmkteq	(1)	1								
bmw	(2)	−0.3270*	1							
Leveragew	(3)	−0.1652*	0.2878*	1						
netlevw	(4)	−0.1156*	0.2385*	0.9811*	1					
ret1yw	(5)	0.0989*	−0.2481*	−0.1675*	−0.1414*	1				
rmedinfw	(6)	−0.1552*	0.1997*	0.1830*	0.1780*	−0.1941*	1			
avgturdw	(7)	0.0098	0.0061	0.0668*	0.0344*	−0.0311*	0.0846*	1		
cvturdw	(8)	−0.4842*	0.0494*	0.0419*	0.016	0.0106	0.0251	−0.0428*	1	
dispeps1w	(9)	0.0027	0.1422*	0.2399*	0.2174*	−0.0940*	0.2018*	0.1566*	0.0071	1
rd	(10)	−0.1589*	−0.1453*	−0.0943*	−0.1292*	−0.0369*	−0.1509*	0.0763*	0.1969*	0.0312*
ad	(11)	0.0011	−0.0599*	−0.0258*	−0.0232	−0.0077	−0.0134	0.0289*	0.0183	−0.0629*
capex	(12)	0.0334*	−0.0104	0.0176	0.0383*	−0.0223	0.0451*	0.0360*	−0.0355*	0.1201*
investmentw	(13)	−0.1219*	−0.1642*	−0.0872*	−0.1066*	−0.0630*	−0.1159*	0.1167*	0.1474*	0.0713*
expgrthw	(14)	−0.1373*	−0.0534*	−0.0782*	−0.0788*	0.0645*	0.4761*	0.0756*	0.0974*	−0.0636*
zscorew	(15)	0.0751*	−0.1825*	−0.1801*	−0.1709*	0.1009*	−0.0788*	0.0728*	−0.0193	−0.1247*
displtg	(16)	−0.0752*	0.0412*	0.0124	−0.0099	0.0152	0.0942*	0.2116*	0.0187	0.0671*
sdroa5yw	(17)	−0.2828*	−0.1043*	−0.0646*	−0.1039*	−0.0202	−0.0244	0.2176*	0.2489*	0.1114*
inv_basew	(18)	0.0807*	−0.0079	0.0058	0.0191	0.0072	−0.0398*	−0.0874*	−0.0385*	0.0442*

		(10)	(11)	(12)	(13)	(14)	(15)	(16)	(17)	(18)
lnmkteq	(1)									
bmw	(2)									
Leveragew	(3)									
netlevw	(4)									
ret1yw	(5)									
rmedinfw	(6)									
avgturdw	(7)									
cvturdw	(8)									
dispeps1w	(9)									
rd	(10)	1								
ad	(11)	−0.0638*	1							
capex	(12)	−0.0894*	0.0021	1						
investmentw	(13)	0.6811*	0.3063*	0.3870*	1					
expgrthw	(14)	0.0003	−0.008	0.0473*	0.0317*	1				
zscorew	(15)	−0.0992*	0.0530*	−0.0801*	−0.0439*	0.1329*	1			
displtg	(16)	0.1132*	−0.0520*	0.0637*	0.0912*	0.3545*	0.015	1		
sdroa5yw	(17)	0.4413*	0.0233	0.0329*	0.4313*	0.1105*	0.0526*	0.2084*	1	
inv_basew	(18)	−0.0616*	0.0379*	0.0319*	−0.0219	−0.0704*	0.0105	−0.0713*	−0.0898*	1

This table presents the correlation coefficients among the explanatory variables for all firms (except financial and utility firms) covered by KLD between 1991 and 2012. All variables are defined in Table 1. * Statistical significance at the 1 % level (*p* < 0.01)

### Multivariate Analysis

Panel A of Table 6 reports the results of the regressions when using the aggregate social performance (SP) measure based on three methods: two-way cluster, fixed effects, and instrumental variable (IV) technique.

**Table 6:** Relation between the risk measures and social performance

Panel A	Two-way cluster			Fixed Effects			IV		
	voldw	IV4ffdw	betad4ffw	voldw	IV4ffdw	betad4ffw	voldw	IV4ffdw	betad4ffw
SP	−0.004	−0.018	−0.097	−0.020	−0.004	0.021	0.037	0.082	−0.037
	(−0.15)	(−0.95)	(−1.36)	(−1.03)	(−0.24)	(0.32)	(0.35)	(0.85)	(−0.10)
SP_crisis	−0.138**	−0.029	−0.082	−0.201***	−0.102**	−0.170	−0.159	−0.114	−0.119
	(−2.38)	(−0.77)	(−0.45)	(−3.89)	(−2.56)	(−1.48)	(−1.37)	(−1.12)	(−0.34)
SP_postcrisis	−0.010	0.032	0.164*	0.016	0.036**	0.087	−0.052	−0.069	0.106
	(−0.31)	(1.12)	(1.72)	(0.73)	(2.01)	(1.26)	(−0.48)	(−0.71)	(0.30)
lnmkteq	−0.034***	−0.033***	−0.034***	−0.046***	−0.046***	−0.007	−0.035***	−0.033***	−0.033***
	(−8.25)	(−9.72)	(−7.93)	(−14.92)	(−17.20)	(−0.84)	(−26.49)	(−28.81)	(−10.80)
bmw	0.022**	0.017**	0.028**	0.012**	0.009*	−0.004	0.022***	0.016***	0.029***
	(2.47)	(2.04)	(1.97)	(2.22)	(1.66)	(−0.31)	(4.80)	(3.73)	(2.59)
netlevw	0.032***	0.030***	0.024**	0.033***	0.032***	0.022***	0.032***	0.030***	0.023***
	(8.61)	(8.96)	(2.54)	(12.31)	(12.76)	(3.77)	(14.40)	(14.36)	(4.50)
rmedinfw	0.119	0.130*	0.304*	0.103**	0.136***	0.181	0.118***	0.130***	0.347***
	(1.37)	(1.78)	(1.94)	(2.48)	(3.68)	(1.52)	(2.94)	(3.57)	(3.22)
avgturdw	0.026***	0.022***	0.025***	0.031***	0.025***	0.020***	0.025***	0.021***	0.024***
	(10.37)	(9.96)	(5.31)	(28.45)	(27.39)	(7.09)	(29.70)	(28.85)	(11.68)
cvturdw	1.225***	1.715***	−2.246***	0.962***	1.375***	−1.770***	1.251***	1.746***	−2.248***
	(12.59)	(13.67)	(−9.86)	(15.70)	(23.13)	(−11.16)	(18.00)	(26.22)	(−13.55)

Panel A	Two-way cluster			Fixed Effects			IV		
	voldw	IV4ffdw	betad4ffw	voldw	IV4ffdw	betad4ffw	voldw	IV4ffdw	betad4ffw
dispeps1w	0.009	0.010	0.071*	0.080***	0.066***	0.029	0.016	0.018*	0.069**
	(0.56)	(0.77)	(1.71)	(6.78)	(6.40)	(0.89)	(1.41)	(1.78)	(2.12)
investmentw	0.157***	0.146***	0.172***	0.045**	0.027	0.128**	0.154***	0.140***	0.168***
	(4.26)	(5.06)	(2.74)	(2.17)	(1.45)	(2.14)	(9.62)	(10.06)	(3.89)
expgrthw	0.069***	0.046***	0.090**	0.041***	0.021**	0.037	0.069***	0.046***	0.083***
	(3.51)	(3.24)	(2.53)	(3.86)	(2.28)	(1.32)	(6.54)	(4.81)	(3.07)
zscorew	0.000	0.000	−0.001	0.003***	0.002***	0.002**	0.000	0.000	−0.001**
	(0.76)	(0.73)	(−1.38)	(7.97)	(6.43)	(2.07)	(1.03)	(0.56)	(−1.96)
sdroa5yw	0.447***	0.423***	0.331***	0.366***	0.281***	0.304***	0.439***	0.416***	0.315***
	(8.99)	(11.17)	(3.10)	(11.15)	(10.44)	(3.07)	(18.31)	(19.71)	(5.17)
inv_basew	−0.031***	−0.024***	−0.049***	−0.035***	−0.030***	−0.037*	−0.031***	−0.024***	−0.055***
	(−5.09)	(−4.84)	(−3.62)	(−4.98)	(−4.96)	(−1.76)	(−5.97)	(−5.43)	(−4.31)
Constant	0.405***	0.372***	1.309***	0.548***	0.509***	1.282***	0.410***	0.378***	1.323***
	(10.59)	(12.47)	(16.80)	(24.27)	(25.70)	(19.75)	(14.59)	(15.44)	(16.29)
Industry fixed effects	Yes	Yes	Yes				Yes	Yes	Yes
Year fixed effects	Yes	Yes	Yes	Yes	Yes	Yes	Yes	Yes	Yes
Firm fixed effects				Yes	Yes	Yes			
Observations	23,423	23,423	23,423	23,423	23,423	23,423	21,730	21,730	21,730
*R* squared	0.682	0.656	0.177	0.651	0.592	0.034	0.683	0.656	0.173
Number of firms	3420	3420	3420	3420	3420	3420	3280	3280	3280
*F* statistic *p* value							0	0	0
*J* statistic *p* value							0.691	0.445	0.390
*p* value (SP + SP_crisis)	0.011	0.170	0.251	0.000	0.008	0.173	0.021	0.440	0.157
*p* value (SP + SP_postcrisis)	0.665	0.539	0.201	0.708	0.001	0.002	0.205	0.210	0.049

Panel B	Two-way cluster			Fixed Effects			IV		
	voldw	IV4ffdw	betad4ffw	voldw	IV4ffdw	betad4ffw	voldw	IV4ffdw	betad4ffw
Str	0.193***	0.184***	−0.070	−0.011	0.014	−0.003	0.553*	0.617**	−2.087**
	(3.32)	(3.82)	(−0.59)	(−0.33)	(0.50)	(−0.03)	(1.86)	(2.34)	(−2.16)
Con	0.129***	0.146***	0.113	0.021	0.013	−0.038	0.103	0.080	−0.392
	(4.43)	(5.19)	(1.16)	(0.92)	(0.68)	(−0.50)	(0.97)	(0.87)	(−1.07)
Str_crisis	−0.402***	−0.328***	−0.205	−0.358***	−0.285***	−0.267**	−0.672**	−0.682***	1.443*
	(−7.35)	(−6.70)	(−0.91)	(−6.19)	(−6.48)	(−2.23)	(−2.56)	(−2.94)	(1.72)
Str_postcrisis	−0.179***	−0.137***	0.142	−0.004	0.012	0.095	−0.510*	−0.542**	1.953**
	(−4.19)	(−3.89)	(1.08)	(−0.14)	(0.45)	(1.00)	(−1.85)	(−2.21)	(2.17)
Con_crisis	−0.035	−0.169***	−0.003	0.081	−0.035	0.095	0.008	−0.080	0.257
	(−0.45)	(−3.82)	(−0.01)	(1.39)	(−0.79)	(0.67)	(0.07)	(−0.82)	(0.75)
Con_postcrisis	−0.071**	−0.093***	−0.152	−0.030	−0.037	−0.097	−0.024	−0.007	0.236
	(−2.28)	(−3.36)	(−1.47)	(−1.09)	(−1.58)	(−1.06)	(−0.24)	(−0.08)	(0.69)
lnmkteq	−0.038***	−0.037***	−0.034***	−0.046***	−0.046***	−0.007	−0.043***	−0.042***	−0.001
	(−8.25)	(−10.05)	(−6.50)	(−14.88)	(−17.18)	(−0.80)	(−10.98)	(−12.38)	(−0.09)
bmw	0.019**	0.013	0.027**	0.011**	0.008	−0.005	0.016***	0.010*	0.051***
	(2.05)	(1.58)	(2.02)	(2.07)	(1.46)	(−0.33)	(2.97)	(1.94)	(3.63)
netlevw	0.031***	0.029***	0.024**	0.033***	0.032***	0.022***	0.030***	0.028***	0.029***
	(8.32)	(8.53)	(2.55)	(12.31)	(12.78)	(3.77)	(11.98)	(11.80)	(4.81)
rmedinfw	0.118	0.129*	0.306*	0.109***	0.142***	0.186	0.113***	0.123***	0.369***
	(1.36)	(1.75)	(1.94)	(2.62)	(3.82)	(1.56)	(2.83)	(3.44)	(3.35)
avgturdw	0.027***	0.022***	0.025***	0.031***	0.026***	0.020***	0.027***	0.022***	0.019***
	(10.64)	(10.31)	(5.31)	(28.43)	(27.44)	(7.11)	(26.92)	(26.62)	(7.13)
cvturdw	1.205***	1.693***	−2.249***	0.968***	1.379***	−1.765***	1.217***	1.710***	−2.107***
	(12.33)	(13.41)	(−9.60)	(15.79)	(23.21)	(−11.14)	(17.06)	(25.23)	(−12.04)

Panel B	Two-way cluster			Fixed Effects			IV		
	voldw	IV4ffdw	betad4ffw	voldw	IV4ffdw	betad4ffw	voldw	IV4ffdw	betad4ffw
dispeps1w	0.004	0.006	0.070*	0.079***	0.065***	0.028	0.009	0.011	0.092***
	(0.29)	(0.47)	(1.70)	(6.66)	(6.28)	(0.87)	(0.78)	(1.03)	(2.64)
investmentw	0.152***	0.141***	0.171***	0.044**	0.026	0.127**	0.140***	0.127***	0.218***
	(4.10)	(4.89)	(2.73)	(2.13)	(1.38)	(2.13)	(8.17)	(8.38)	(4.34)
expgrthw	0.070***	0.047***	0.090**	0.040***	0.021**	0.037	0.070***	0.047***	0.077***
	(3.56)	(3.31)	(2.52)	(3.83)	(2.25)	(1.31)	(6.68)	(5.01)	(2.78)
zscorew	0.000	0.000	−0.001	0.003***	0.002***	0.002**	0.000	0.000	−0.002***
	(0.96)	(0.99)	(−1.36)	(7.98)	(6.48)	(2.08)	(1.55)	(1.28)	(−2.58)
sdroa5yw	0.435***	0.411***	0.329***	0.367***	0.281***	0.306***	0.418***	0.393***	0.390***
	(8.78)	(10.78)	(3.08)	(11.17)	(10.47)	(3.09)	(16.54)	(17.75)	(5.76)
inv_basew	−0.034***	−0.027***	−0.049***	−0.036***	−0.031***	−0.038*	−0.038***	−0.031***	−0.030*
	(−5.42)	(−5.29)	(−3.78)	(−5.06)	(−5.03)	(−1.79)	(−6.25)	(−5.93)	(−1.77)
Constant	0.426***	0.394***	1.310***	0.545***	0.507***	1.278***	0.452***	0.422***	1.160***
	(10.88)	(13.29)	(16.23)	(24.13)	(25.54)	(19.57)	(15.36)	(17.12)	(9.27)
Industry fixed effects	Yes	Yes	Yes				Yes	Yes	Yes
Year fixed effects	Yes	Yes	Yes	Yes	Yes	Yes	Yes	Yes	Yes
Firm fixed effects				Yes	Yes	Yes			
Observations	23,423	23,423	23,423	23,423	23,423	23,423	21,730	21,730	21,730
*R* squared	0.685	0.660	0.177	0.651	0.593	0.034	0.682	0.654	0.134
Number of firms	3420	3420	3420	3420	3420	3420	3280	3280	3280
F statistic *p* value							0	0	0
*J* statistic *p* value							0.384	0.208	0.308
*p* value (Str + Str_crisis)	0.011	0.037	0.161	0.000	0.000	0.036	0.103	0.290	0.001
*p* value (Str + Str_postcrisis)	0.678	0.105	0.243	0.268	0.027	0.027	0.107	0.001	0.114
*p* value (Con + Con_crisis)	0.152	0.550	0.448	0.082	0.615	0.680	0.091	0.999	0.405
*p* value (Con + Con_postcrisis)	0.021	0.001	0.542	0.691	0.208	0.074	0.002	0.001	0.053

Columns 2–4 of Table 6 reports results from two-way cluster regressions of the risk measures on the social performance measures and controls over the period 1991–2012. Robust and clustered (by firm and time) *t* statistics based on the approach of Petersen (2009) are reported in the parentheses. Columns 5–7 of Table 6 reports results from two-way fixed effects (FE) regressions of the risk measures on the social performance measures and controls over the period 1991–2012. Robust and clustered (by firm) *t* statistics are reported in the parentheses. Columns 8–10 of Table 6 reports results from instrumental variables (IV) regressions of the risk measures on the social performance measures and controls over the period 1991–2012. The IV regressions are estimated using the two-step efficient generalized method of moments (GMM). We use two instruments: the average SP of neighboring firms (geographically proximate firms) and the average industry SP. *J* statistic *p* value is the *p* value of the Hansen J statistic (overidentification test of all instruments). Robust and clustered (by firm) *t* statistics are reported in parentheses

The *p* value (SP + SP_crisis) is the *p* value of the test statistic that the sum of the coefficients associated with SP and SP_crisis is not statistically different from zero. The *p* value (SP + SP_postcrisis) is the *p* value of the test statistic that the sum of the coefficients associated with SP and SP_postcrisis is not statistically different from zero. The *p* value (Str + Str_crisis) is the *p* value of the test statistic that the sum of the coefficients associated with Str and Str_crisis is not statistically different from zero. The *p* value (Str + Str_postcrisis) is the *p* value of the test statistic that the sum of the coefficients associated with Str and Str_postcrisis is not statistically different from zero. The *p* value (Con + Con_crisis) is the *p* value of the test statistic that the sum of the coefficients associated with Con and Con_crisis is not statistically different from zero. The *p* value (Con + Con_postcrisis) is the *p* value of the test statistic that the sum of the coefficients associated with Con and Con_postcrisis is not statistically different from zero

All variables are defined in Table 1. Unreported industry controls are based on the Fama and French (1997) industry classification

*** Significant at the 1 % level (*p* < 0.01); ** Significant at the 5 % level (*p* < 0.05); * Significant at the 10 % level (*p* < 0.1)

Panel A of Table 6 shows that the coefficient associated with SP, which captures the impact of SP on risk in the pre-crisis period, is insignificant regardless of the methodology used. Thus, SP seems to have no effect on risk in the pre-crisis period. However, the variable SP_crisis is significantly and negatively related to the dependent variable, stock return volatility, in two of the three methodologies (two-way cluster and fixed effects). The *p* value of the sum $(\alpha_{1} + \alpha_{2} )$ is lower than 5 % in both cases, which implies that $(\alpha_{1} + \alpha_{2} )$ is significantly different from zero. The *p* value of the sum $(\alpha_{1} + \alpha_{2} )$ is also lower than 5 % under the IV method, although the coefficients are not significant individually. Thus, the evidence suggests that SP reduces stock return volatility significantly during the financial crisis. In terms of economic significance, an increase in SP by one standard deviation during the financial crisis decreases the firm’s volatility by about 1.18–1.84 %.^31^

The variable SP_crisis is also significantly and negatively related to the dependent variable, idiosyncratic risk, in one of the three methodologies (fixed effects). The *p* value of the sum $(\alpha_{1} + \alpha_{2} )$ is lower than 5 %, which implies that $(\alpha_{1} + \alpha_{2} )$ is significantly different from zero. In terms of economic significance, an increase in SP by one standard deviation decreases the firm’s idiosyncratic risk by about 1.14 % during the financial crisis. This evidence suggesting that SP reduces idiosyncratic risk during the financial crisis is rather weak since it holds only in one of the three methodologies. The coefficient associated with SP_postcrisis is positive and significant when idiosyncratic risk is the dependent variable in one of the three methodologies (fixed effects). This increase in idiosyncratic risk after the financial crisis (measured by the sum $(\alpha_{1} + \alpha_{3} )$) is 1.24 %, which is statistically significant (*p* value of the sum $(\alpha_{1} + \alpha_{3} )$ < 5 %). The coefficients associated with SP_crisis and SP_postcrisis are insignificant when systematic risk is the dependent variable.

Panel B of Table 6 shows that the coefficients of the aggregate measures of strengths (Str) and concerns (Con) are positive and statistically significant when the dependent variable is either total or idiosyncratic risk. However, those effects are not consistent across the different methodologies (hold only in one out of three methodologies), except for the coefficient of the aggregate measure of strengths (Str) which is significant in two of the three methodologies (two-way cluster and IV) when the dependent variable is idiosyncratic risk. In terms of economic significance, an increase in the aggregate measure of strengths (Str) by one standard deviation before the financial crisis increases the firm’s idiosyncratic risk by about 1.65–5.55 %. Besides, the coefficient of the aggregate measure of strengths (Str) is negative and statistically significant when the dependent variable is systematic risk in only one of the three methodologies (IV). Moreover, the effect on systematic risk becomes insignificant when beta is measured using the CAPM model.^32^

As shown in Panel B of Table 6, the variable Str_crisis is significantly and negatively related to the dependent variable, stock return volatility, regardless of the methodology used. Thus, the evidence suggests that the sensitivity of volatility to changes in the aggregate measure of strengths (Str) becomes significantly negative during the financial crisis. In terms of economic significance, an increase in the aggregate measure of strengths (Str) by one standard deviation decreases the firm’s volatility by about 0.83–2.57 % during the financial crisis. This effect is based on the sum $(\alpha_{11} + \alpha_{12} )$ and its associated *p* values.

The variable Str_crisis is also significantly and negatively related to the dependent variable, idiosyncratic risk, regardless of the methodology used. The evidence suggests that the aggregate measure of strengths (Str) reduces idiosyncratic risk significantly during the financial crisis. In terms of economic significance, an increase in the aggregate measure of strengths (Str) by one standard deviation decreases the firm’s idiosyncratic risk by about 0.58–2.43 % during the financial crisis. This effect is based on the sum $(\alpha_{11} + \alpha_{12} )$ and its associated *p* values.

The coefficient associated with Str_crisis is insignificant when systematic risk is the dependent variable in two out of three methods suggesting that the aggregate measure of strengths (Str) did not change the relation between Str and systematic risk during the financial crisis.^33^ However, the variable Str_postcrisis is significantly and positively related to the dependent variable, systematic risk, in one of the three methodologies (IV). The coefficient associated with Str_postcrisis is negative and significant when total risk is the dependent variable in one of the three methodologies (two-way cluster). In terms of economic significance, a one standard deviation change in Str during the post-crisis period decreases systematic risk by about 1.57 %, but increases total risk by about 0.39 %. Those effects are not significant as the *p* values of the sum of the coefficients for Str and Str_postcrisis $(\alpha_{11} + \alpha_{13} )$ are higher than 10 %.

The variable Str_postcrisis is significantly and negatively related to the dependent variable, idiosyncratic risk, in two of the three methodologies (two-way cluster and IV). The *p* value of the sum $(\alpha_{11} + \alpha_{13} )$ is lower than 5 % when using the IV method, but higher than 10 % when using the two-way cluster method. Thus, there is weak evidence suggesting that the aggregate measure of strengths (Str) reduces idiosyncratic risk significantly after the financial crisis.

Panel B of Table 6 also reports the results of the potential impact of the aggregate measure of concerns (Con) on a firm’s risk during and after the financial crisis. The results show much less statistical significance suggesting a lower or no impact at all. There is some evidence suggesting that the sensitivities of volatility and idiosyncratic risk (when using the two-way cluster method) to the aggregate measure of concerns (Con) have decreased after the financial crisis. In terms of economic significance, a one standard deviation change in Con after the crisis period based on the sum $(\alpha_{21} + \alpha_{23} )$ using the two-way cluster method translates into an increase of 0.98 % in volatility and 1.14 % in idiosyncratic risk. With one exception (when using the two-way cluster method and the dependent variable is idiosyncratic risk), none of the coefficients associated with Con_crisis is significant suggesting that the aggregate measure of concerns (Con) has no impact on a firm’s risk during the crisis period.

In summary, the results reported in Table 6 have three implications. First, the relation between SP and risk varies over time (is dynamic) and depends on market conditions. Our results indicate that the relation between SP and risk is significantly different in the crisis period compared to the pre-crisis period. Second, social performance reduces volatility and idiosyncratic risk significantly during the financial crisis, primarily due to the strengths component of SP. However, the impact of SP on systematic risk during the financial crisis is less obvious. Third, the relation between the strengths and risk is stronger than the relation between the concerns and risk during the financial crisis, which suggests an asymmetric relation between the SP components and a firm’s risk. During the financial crisis, the magnitude and statistical significance of the coefficients associated with the aggregate measure of strengths (Str) are higher than those of the coefficients associated with the aggregate concerns measure (Con). This is a new finding as previous CSR findings reported in the literature that the asymmetric financial effect is due to concerns, not strengths (e.g., Kappou and Oikonomou 2014, in a different context).^34^ In particular, we show that strengths are very useful in terms of risk reduction during tough periods (e.g., financial crises or economic recessions). This implies that firms belonging to the “lowtier” or “zerotier” group could benefit by migrating to the other two groups (i.e., “medtier” or “toptier” group) if their financial fragility is adversely affected by bad economic conditions.

## Robustness Checks

### Reverse Causality: Simultaneous Equation Framework

Endogeneity bias is a crucial challenge in the CSR empirical literature since it prevents researchers from drawing causal inferences (Jiraporn et al. 2014). In the previous section, we controlled for the endogeneity of SP using the instrumental variable (IV) approach. However, another potential source of endogeneity is *simultaneity bias* if SP and the firm’s risk are jointly determined. The risk mitigation view (i.e., the stakeholder theory perspective) predicts that firms with higher SP could have higher financial performance (e.g., due to lower risk). However, there exist theoretical justifications for the proposition that financial performance causes SP (e.g., slack resources hypothesis).^35^ Waddock and Graves (1997) find that SP is both a predictor and consequence of financial performance. That is, there is a simultaneous relationship, or a kind of ‘virtuous circle’, which they explain by a simultaneous and interactive impact between theoretical arguments such as the slack resources theory and the stakeholder theory. In our context, this implies that a firm’s risk may in turn affect its SP in several ways. For example, the largest firms with rather stable cash flows, and generally lower stock price volatility, can afford to initiate social actions. All else held equal, lower cash flow volatility and enduring profitability are prerequisites for social commitment according to the slack resources hypothesis. It is also possible that managers of less risky firms may be less prone to improve their SP due to lower stakeholders’ pressure. Alternatively, managers of risky firms may improve SP in an attempt to change the perceptions of investors and analysts about the risk profile of their firms. Based on a meta-analysis of 18 studies that examine the relationship between SP and firm risk in any form, Orlitzky and Benjamin (2001) find that prior SP is negatively related to subsequent firm risk, and prior firm risk is negatively related to subsequent SP.

To address this particular form of endogeneity, we follow Mishra and Modi (2013) and use a *simultaneous equations system* where SP affects the firm’s risk and is, in turn, affected by the latter. Specifically, we estimate a simultaneous system of equations using three-stage-least squares (3SLS):

8a $${\text{Str}}_{it} = \beta_{0} + \lambda_{1} {\text{Risk}}_{it - 1} + \pi_{1} {\text{Str}}_{it - 1} + \varphi_{1} {\text{Con}}_{it - 1} + \theta X_{it - 1} + \gamma_{i} + \eta_{t} + \omega_{it}$$

8b $${\text{Con}}_{it} = \upsilon_{0} + \lambda_{2} {\text{Risk}}_{it - 1} + \pi_{2} {\text{Str}}_{it - 1} + \varphi_{2} {\text{Con}}_{it - 1} + \delta X_{it - 1} + \gamma_{i} + \eta_{t} + \mu_{it}$$

8c $${\text{Risk}}_{it} = \alpha_{0} + \alpha_{11} {\text{Str}}_{it} + \alpha_{12} {\text{Str}}_{it} \times {\text{Crisis}}_{it} + \alpha_{13} {\text{Str}}_{it} \times {\text{PostCrisis}}_{it} + \alpha_{21} {\text{Con}}_{it} + \alpha_{22} {\text{Con}}_{it} \times {\text{Crisis}}_{it} + \alpha_{23} {\text{Con}}_{it} \times {\text{PostCrisis}}_{it} + \psi X_{it} + \gamma_{i} + \eta_{t} + \varepsilon_{it}$$

where ${\text{Risk}}_{it}$ is the risk measure and ${\text{Str}}_{it}$ (${\text{Con}}_{it}$) is the strengths (concerns) measure for firm *i* at time *t*. The three equations of the system have the same set of control variables. ${\text{Risk}}_{it}$, ${\text{Str}}_{it}$, and ${\text{Con}}_{it}$ are now treated as being endogenous. Following Mishra and Modi (2013), we include firms’ fixed effects to control for unobserved time invariant firm characteristics (*γ*_*i*_). Since our sample includes more than 3000 firms, it is not convenient to include thousands of dummies in the system of equations. Instead, we first remove the fixed effects from all variables, including dependent and independent variables. This is achieved by demeaning all the variables, i.e., for every company, we subtract the mean value of the variable across time from each observation. Then, we estimate the system of equations using the 3SLS method applied on the demeaned variables. We also include time dummies in all equations (*η*_*t*_).

The results of the 3SLS estimation reported in Table 7 are consistent with the results reported in Table 6. The coefficient associated with the variable Str_crisis is negative and statistically significant when either volatility or idiosyncratic risk is the dependent variable. Thus, the evidence suggests that the sensitivity of volatility and idiosyncratic risk to changes in the aggregate measure of strengths (Str) changes significantly and becomes negative during the financial crisis. In terms of economic significance, an increase in the aggregate measure of strengths (Str) by one standard deviation decreases the firm’s volatility (idiosyncratic risk) by about 1.74 % (1.88 %) during the financial crisis based on the sum $(\alpha_{11} + \alpha_{12} )$ and its associated *p* value.

**Table 7:** 3SLS regressions between the risk measures and social performance measures

	voldw	Str	Con	IV4ffdw	Str	Con	betad4ffw	Str	Con
Risk		−0.015***	0.010***		−0.016***	0.005		0.000	−0.002**
		(−4.24)	(3.71)		(−3.99)	(1.46)		(0.22)	(−2.10)
Str	0.172	0.684***	−0.039***	0.181*	0.684***	−0.039***	0.268	0.685***	−0.039***
	(1.47)	(106.35)	(−7.54)	(1.78)	(106.32)	(−7.62)	(0.71)	(106.43)	(−7.56)
Con	0.036	0.068***	0.456***	0.075	0.067***	0.457***	−0.267	0.068***	0.457***
	(0.62)	(8.76)	(73.03)	(1.50)	(8.58)	(73.04)	(−1.44)	(8.71)	(73.06)
Str_crisis	−0.423***			−0.391***			−0.470**		
	(−6.12)			(−6.53)			(−2.10)		
Str_postcrisis	−0.156			−0.151*			−0.275		
	(−1.57)			(−1.75)			(−0.85)		
Con_crisis	0.244***			−0.009			0.001		
	(5.28)			(−0.22)			(0.01)		
Con_postcrisis	0.025			−0.092**			−0.203		
	(0.53)			(−2.22)			(−1.31)		
lnmkteq	−0.046***	0.008***	0.009***	−0.049***	0.007***	0.008***	−0.015***	0.008***	0.008***
	(−26.17)	(9.20)	(13.15)	(−32.14)	(8.95)	(12.64)	(−2.62)	(10.30)	(12.61)
bmw	0.017***	0.009***	0.007***	0.007***	0.008***	0.007***	−0.011	0.008***	0.007***
	(5.77)	(5.58)	(5.93)	(2.73)	(5.51)	(6.07)	(−1.16)	(5.29)	(5.74)
netlevw	0.034***	0.001*	0.002***	0.033***	0.001**	0.002***	0.015***	0.001	0.002***
	(29.53)	(1.88)	(3.60)	(33.49)	(2.02)	(4.13)	(4.12)	(0.80)	(4.04)
rmedinfw	0.140***	0.096***	0.012	0.126***	0.097***	0.014	0.297***	0.093***	0.013
	(5.44)	(7.53)	(1.18)	(5.64)	(7.62)	(1.36)	(3.56)	(7.27)	(1.26)

	voldw	Str	Con	IV4ffdw	Str	Con	betad4ffw	Str	Con
avgturdw	0.030***	0.003***	0.001***	0.024***	0.003***	0.001***	0.017***	0.002***	0.002***
	(47.97)	(8.99)	(4.29)	(43.53)	(8.74)	(5.05)	(8.06)	(8.17)	(6.27)
cvturdw	0.995***	−0.005	−0.021	1.463***	0.006	−0.017	−1.631***	−0.015	−0.013
	(23.27)	(−0.25)	(−1.29)	(39.45)	(0.30)	(−1.00)	(−11.80)	(−0.76)	(−0.81)
dispeps1w	0.081***	−0.006	0.011***	0.064***	−0.006	0.011***	0.026	−0.007*	0.011***
	(10.55)	(−1.52)	(3.54)	(9.56)	(−1.54)	(3.66)	(1.05)	(−1.94)	(3.64)
investmentw	0.046***	−0.003	−0.003	0.044***	−0.003	−0.002	0.152***	−0.005	−0.004
	(3.29)	(−0.50)	(−0.56)	(3.66)	(−0.43)	(−0.42)	(3.36)	(−0.81)	(−0.71)
expgrthw	0.030***	−0.004	−0.008***	0.019***	−0.004	−0.008***	0.001	−0.005	−0.008***
	(4.09)	(−0.99)	(−2.84)	(2.87)	(−1.05)	(−2.75)	(0.03)	(−1.29)	(−2.84)
zscorew	0.002***	−0.000***	−0.000***	0.002***	−0.000***	−0.000***	0.003***	−0.000***	−0.000***
	(9.50)	(−3.98)	(−4.28)	(8.54)	(−4.01)	(−4.02)	(4.01)	(−4.47)	(−3.99)
sdroa5yw	0.327***	0.006	0.025***	0.254***	0.005	0.027***	0.298***	0.001	0.030***
	(15.60)	(0.64)	(3.17)	(13.99)	(0.54)	(3.49)	(4.40)	(0.07)	(3.83)
inv_basew	−0.039***	−0.008***	−0.005**	−0.024***	−0.008***	−0.005**	−0.038**	−0.008***	−0.005**
	(−7.11)	(−3.26)	(−2.33)	(−5.11)	(−3.18)	(−2.45)	(−2.14)	(−3.01)	(−2.53)
Constant	0.130***	−0.004	0.001	0.042***	−0.003	−0.006***	0.044***	−0.005**	−0.005***
	(28.48)	(−1.64)	(0.42)	(10.58)	(−1.52)	(−3.11)	(2.95)	(−2.47)	(−2.73)
Firm fixed effects	Yes	Yes	Yes	Yes	Yes	Yes	Yes	Yes	Yes
Year fixed effects	Yes	Yes	Yes	Yes	Yes	Yes	Yes	Yes	Yes
Observations	18,994	18,994	18,994	18,994	18,994	18,994	18,994	18,994	18,994
*R* squared	0.645	0.501	0.424	0.584	0.501	0.424	0.026	0.500	0.424
F statistic *p* value	0	0	0	0	0	0	0	0	0
*p* value (Str + Str_crisis)	0.000			0.000			0.456		
*p* value (Str + Str_postcrisis)	0.482			0.130			0.921		
*p* value (Con + Con_crisis)	0.000			0.062			0.044		
*p* value (Con + Con_postcrisis)	0.025			0.456			0.000		

Table 7 report results from the 3SLS regressions of the simultaneous equations system where the dependent variables are the risk, strengths, and concerns measures over the period 1991–2012. The simultaneous system of equations estimated using three-stage-least squares (3SLS) with all terms defined in Table 1 is

$${\text{Str}}_{it} = \beta_{0} + \lambda_{1} {\text{Risk}}_{it - 1} + \pi_{1} {\text{Str}}_{it - 1} + \varphi_{1} {\text{Con}}_{it - 1} + \theta X_{it - 1} + \gamma_{i} + \eta_{t} + \omega_{it}$$

$${\text{Con}}_{it} = \upsilon_{0} + \lambda_{2} {\text{Risk}}_{it - 1} + \pi_{2} {\text{Str}}_{it - 1} + \varphi_{2} {\text{Con}}_{it - 1} + \delta X_{it - 1} + \gamma_{i} + \eta_{t} + \mu_{it}$$

$$\begin{aligned} {\text{Risk}}_{it} = \alpha_{0} + \alpha_{11} {\text{Str}}_{it} + \alpha_{12} {\text{Str}}_{it} \times {\text{Crisis}}_{it} + \alpha_{13} {\text{Str}}_{it} \times {\text{PostCrisis}}_{it} + \alpha_{21} {\text{Con}}_{it} \hfill \\ +\, \alpha_{22} {\text{Con}}_{it} \times {\text{Crisis}}_{it} + \alpha_{23} {\text{Con}}_{it} \times {\text{PostCrisis}}_{it} + \psi X_{it} + \gamma_{i} + \eta_{t} + \varepsilon_{it} \hfill \\ \end{aligned}$$

We first remove the fixed effects from all variables, including dependent and independent variables. This is achieved by demeaning all the variables, i.e., for every company, we subtract the mean value of the variable across time from each observation. Then, we estimate the system of equations using the 3SLS method applied on the demeaned variables. We also included time dummies in all equations. The *p* value (Str + Str_crisis) is the *p* value of the test statistic that the sum of the coefficients associated with Str and Str_crisis is not statistically different from zero. The *p* value (Str + Str_postcrisis) is the *p* value of the test statistic that the sum of the coefficients associated with Str and Str_postcrisis is not statistically different from zero. The *p* value (Con + Con_crisis) is the *p* value of the test statistic that the sum of the coefficients associated with Con and Con_crisis is not statistically different from zero. The *p* value (Con + Con_postcrisis) is the *p* value of the test statistic that the sum of the coefficients associated with Con and Con_postcrisis is not statistically different from zero. All variables are defined in Table 1. Robust *t* statistics are reported in the parentheses

*** Significant at the 1 % level (*p* < 0.01); ** Significant at the 5 % level (*p* < 0.05); * Significant at the 10 % level (*p* < 0.1)

When the systematic risk is the dependent variable, the coefficient associated with Str_crisis is also negative and significant. Although statistically significant on its own, the total effect on systematic risk in the crisis period, $(\alpha_{11} + \alpha_{12} )$, is not statistically significant (*p* value = 0.456). Moreover, none of the coefficients associated with Str_postcrisis is significant confirming our results reported in Table 6 where the aggregate measure of strengths (Str) has little or no impact on a firm’s risk after the crisis period.

The results reported in Table 7 also show that the coefficient associated with Con_postcrisis is negative and significant when the dependent variable is idiosyncratic risk as it was the case in Table 6 (using the two-way cluster method). Although statistically significant on its own, the total effect on idiosyncratic risk after the crisis period, $(\alpha_{21} + \alpha_{23} )$, is not statistically significant (*p* value = 0.456). However, Table 7 shows some evidence suggesting that the sensitivity of total risk to changes in the aggregate measure of concerns (Con) has increased during the financial crisis but not thereafter. In terms of economic significance, a one standard deviation change in Con during the crisis period translates into an increase of 2.34 % in volatility based on the sum $(\alpha_{21} + \alpha_{22} )$.

### Alternative Model Specification^36^

We run several sensitivity tests to examine whether our results are robust to alternative model specifications. Specifically, we re-estimate our basic model after replacing and/or adding several control variables. First, we replace expected growth (mean annualized five-year earnings growth rate from I/B/E/S) by average five-year sales growth, and book-to-market ratio by Tobin’s Q. Second, we use Amihud illiquidity measure computed as in Amihud (2002) as an alternative measure of firm liquidity. Third, we use the percentage signed (absolute) forecast error as an alternative measure of earnings variability. Forecast error is measured as the difference between the one-year ahead median earnings forecast and the actual earnings deflated by the stock price at the measurement date of our dependent variables. Fourth, we use two alternative proxies for default risk instead of the *Z*score: bond rating and investment grade rating. Bond Rating is a dummy variable equal to one if the long-term debt of the firm is rated and equal to zero otherwise. Firms without ratings are expected to be more risky than those having ratings. Conditional on having a rating, a firm is categorized as investment grade if it has a rating higher than BB+ and as junk if it has a rating of BB+ or less. Investment grade rating is a dummy variable equal to one if S&P debt rating is higher than BB+ and equal to zero otherwise. Investment grade debt is expected to be less risky than non-investment grade debt. Finally, we include free cash flow to equity (or to the firm) as an additional control variable.^37^ Overall, our untabulated results are robust to all these alternative model specifications.^38^

### Additional Robustness Checks^39^

At first glance, some of our findings seems at odds with those reported in some previous studies (e.g., Mishra and Modi 2013; Oikonomou et al. 2012). For example, some of our results show that both strengths and concerns are positively related to risk before the financial crisis (when using two-way cluster). This seems at odds with those previous studies that found either a negative or insignificant link between strengths and some risk measures.^40^ For example, Mishra and Modi (2013) find that the coefficient for strengths (PCSR) is negative and the one for concerns (NCSR) is positive, and both are highly significant. Similarly, Oikonomou et al. (2012) find that the coefficient for strengths is negatively but weakly related to systematic risk and that the coefficient for concerns is positively and strongly related to systematic risk.

Several factors could explain why our results are somehow different from those previous studies such as differences in sample selection criteria, differences in the empirical design, e.g., different model specifications and estimation techniques, and differences in the time period examined. We now empirically examine each of these three possible explanations. We begin by constructing sub-samples in addition to our original sample which is an unbalanced panel covering the period 1991–2012: a balanced panel covering the period 1991–2012, and a balanced panel covering the period 2000–2009 following Mishra and Modi (2013). We repeat all our empirical analysis for these sub-samples. We discuss below the empirical findings.

Restricting our sample to only those firms with non-missing data in the KLD database over the time period 1991–2012 results in a balanced panel of only 204 firms with a total of 4229 firm-year observations. This balanced sample represents less than 10 % of the full sample (around 3000 firms with a total of 28,110 firm-year observations). Clearly, this sample suffers from severe survivorship and selection biases. Some of the results for this balanced panel are similar to those reported for the full sample, whereas other results are different. For example, the coefficient associated with the variable Str_crisis is negative and statistically significant when either volatility or idiosyncratic risk is the dependent variable. The coefficient associated with the variable Str_postcrisis is also negative and statistically significant. However, the coefficients associated with the variables Con_crisis and Con_postcrisis are insignificant for this balanced panel. Overall, the results for this balanced panel suggest that the relation between the strengths and risk is stronger than the relation between the concerns and risk during and after the financial crisis, which confirm our conjecture regarding the asymmetric relation between the SP components and a firm’s risk.

We now follow Mishra and Modi (2013) by only including firms with non-missing data in the KLD, CRSP, and COMPUSTAT databases over the time period 2000–2009. These sampling criteria result in a balanced panel of only 207 firms with a total of 1742 firm-year observations (Mishra and Modi use a balanced panel of 192 firms with a total of 1728 firm-year observations). As with the previous balanced panel, this sample suffers from severe survivorship and selection biases, e.g., it represents less than 10 % of the full sample covered by KLD. Using their modeling approach (3SLS) as well as the same variables used in their study, we are able to replicate their results. That is, we find that the coefficient associated with strengths (PCSR) is negative and the one associated with concerns (NCSR) is positive, and both are highly significant. We also examine the same sample using their modeling approach (3SLS), but using our variables (those used in our paper). We find similar results. We also examine the same sample and variables as Mishra and Modi (2013), but with different methodologies (two-way cluster regression model of Petersen (2009), the two-way fixed effects model, and the Instrumental variables (IV) regression). Their results hold only in one particular case: the dependent variable is volatility and the estimation method is the fixed effects model. Overall, their results differ depending on the modeling approach used. Clearly, the results of Mishra and Modi (2013) are specific to their sample and the methodology used.

In addition, we examine the impact of using alternative measures of social performance on our results. To do this, we recompute the strengths and concerns measures following Oikonomou et al. (2012). The calculation is similar to the one used in our paper, except that they consider only five dimensions instead of seven. They exclude corporate governance and human rights. The SP measures of Oikonomou et al. (2012) are very highly correlated with our SP measures (0.9 or more) suggesting that the results using any of these measures will not be materially different. We re-estimate our regressions using the SP measures of Oikonomou et al. (2012) and find similar results. The only difference is that the coefficients associated with the variables SP_crisis, Str_crisis, and Str_postcrisis are positive and significant when the dependent variable is beta using the IV method.

Another issue could be the length of the pre-crisis period which is relatively long since it includes 17 years, whereas the post-crisis period includes only 3 years at the end of the sample. Ioannou and Serafeim (2014) have shown that the link between SP and financial performance has strengthened with time. This raises the issue that our findings could be an artifact of that generic trend rather than a response to the crisis itself. We empirically explore this issue by splitting the time-series dimension of the sample in different ways. In particular, we shorten the pre-crisis period by using additional interaction variables in the regressions to see if our results are robust. Specifically, we split the sample into four subperiods: prebubble: 1991–1998 (“the 1990s”), bubble: 1999–2000 (“the internet bubble”), pre-crisis: 2001–2007 (“the 2000s”), crisis: 2008–2009 (“the financial crisis”), and post-crisis: 2010–2012 (“the post-crisis period”). In this way, the coefficient associated with the variable SP (Str or Con) captures the prebubble period (1991–1998). The focus is on the coefficients of the interaction variables associated with the pre-crisis, crisis, and post-crisis. The untabulated results show that the variable Str_crisis continues to be significantly and negatively related to the stock return volatility and idiosyncratic risk in most cases. However, the coefficients associated with the variable Str_precrisis and Con_precrisis are mostly insignificant. Overall, our main findings remain unchanged.

## Conclusion

This paper examines the impact of the recent financial crisis (2008–2009) on the relation between a firm’s risk and social performance (SP) using a sample of non-financial U.S. firms covering the period 1991–2012. The main results can be summarized as follows. First, the relation between SP and risk is time-varying and depends on market conditions. Our results indicate that the relation between SP and risk is significantly different in the crisis period (post-crisis period) compared to the pre-crisis period.

Second, the aggregated social performance or SP (strengths minus concerns) reduces volatility significantly during the financial crisis. An increase in SP of one standard deviation decreases the firm’s volatility by about 1.18–1.84 % during the financial crisis depending upon the estimation method. The risk reduction potential of SP is mainly due to the strengths component of SP. An increase in the aggregate measure of strengths (Str) by one standard deviation decreases the firm’s volatility (idiosyncratic risk) by about 0.83–2.57 % (0.58–2.43 %) during the financial crisis.

Third, the relation between the strengths and risk is stronger than the relation between the concerns and risk during the financial crisis, which suggests an asymmetric relation between the social performance components and a firm’s risk. It follows that strengths are more useful in terms of risk reduction during adverse economic environments (e.g., financial crises, economic recessions).

## Appendix

The expected return is proxied by the cost of equity capital calculated using the implied cost of capital methodology (ICC approach hereafter). The main idea of the ICC approach is to treat each firm as an investment project and to use the valuation equation in order to back out the cost of equity. The cost of equity is the discount rate (or the internal rate of return) that equates the current stock price to the present value of all expected future cash flows. Investors’ expectations are proxied by financial analyst forecasts, assuming that analysts’ forecasts reflect or drive investors’ beliefs. Several studies have used the ICC approach along with forecasted earnings to estimate the cost of equity at the firm-level (e.g., Claus and Thomas 2001; Gebhardt et al. 2001; Easton 2004; Ohlson and Juettner-Nauroth 2005; Hail and Leuz 2006; Witmer and Zorn 2007; Lee et al. 2009). The ICC approach using forecasted earnings is appealing because it provides an *ex ante* cost of equity measure. Most asset pricing theories are formulated in terms of *ex ante* predictions. By inferring the cost of equity from current price and expectations about the future, we can think of the cost of equity as a market-determined measure (Ohlson and Juettner-Nauroth 2005). We follow this research stream by computing the cost of equity for each firm-year observation using five ICC models: PEG ratio model of Easton (2004), MPEG ratio model of Easton (2004), ICC model of Ohlson and Juettner-Nauroth (2005), ICC model of Claus and Thomas (2001), and ICC model of Lee et al. (2009). For each firm-year observation we compute the implied cost of equity using current stock price, book value per share, one-year-ahead and two-year-ahead mean earnings per share forecasts, payout ratio, five-year annualized mean (median) growth rate (an estimate for short-term growth obtained from I/B/E/S), and an estimate for the long-term growth rate (e.g., expected inflation rate). The implementation of the five ICC models is similar to that of Hail and Leuz (2006) and El Ghoul et al. (2011). We use the average implied cost of equity (rmedinfw) based on the five models as our proxy for the cost of equity. Details on the implementation of the five models are available from the authors upon request.

## Footnotes

1 MSCI ESG STATS (former KLD Research & Analytics, Inc). For simplicity, we use the KLD abbreviation.

2
http://www.unpri.org/principles/.

3 The terms CSR and SP are used interchangeably throughout this paper. The concept of SP can be defined as “*a business organization’s configuration of principles of social responsibility, processes of social responsiveness, and policies, programs, and observable outcomes as they relate to the firm’s societal relationships*” (Wood 1991). In broader terms, CSR refers to the extent of relationships between the firm and its key stakeholders (employees, customers, suppliers, community, natural environment, etc.) that go above and beyond what is legally required (Jo and Harjoto 2012). CSR need not be morally motivated (i.e., associated with moral duties), because it could be used strategically to serve the firm’s interests (Jo and Harjoto 2012).

4 Earlier studies use correlation analysis to examine this relationship (e.g., Spicer 1978; McGuire et al. 1988). Based on a meta-analysis of 18 studies that examine the relationship between SP and firm risk in any form, Orlitzky and Benjamin (2001) conclude that SP is negatively correlated with various measures of firm risk, and that the correlation is stronger for market versus accounting measures. More recent studies find that SP is negatively related to the cost of equity capital (Feldman et al. 1997; Sharfman and Fernando 2008; El Ghoul et al. 2011), and financial distress or default risk (Goss 2007).

5 An exception is Luo and Bhattacharya (2009) who examine both idiosyncratic and systematic risks in the same study using a sample of 541 large firms in *Fortune*’s Most Admired Companies over 2 years. They find that higher SP lowers a firm’s idiosyncratic risk for firms with higher versus lower advertising, but that a simultaneous pursuit of SP, advertising, and R&D increases idiosyncratic risk. They also find a negative relationship between systematic risk (CAPM beta) and their SP proxy.

6 This might be attributable to violations of the perfect capital market assumptions in the real world (e.g., costly and risky arbitrage and impediments to full diversification).

7 CSR activities will generate positive moral capital when both the acts themselves and the imputations about the firm receive positive evaluations from stakeholders (Godfrey 2005).

8 Consistent with the “neglect effect” caused by SP, Hong and Kacperczyk (2009) find that sin stocks (tobacco, alcohol and gaming) are neglected by institutional investors subject to social norms such as pension funds. Sin stocks are held less by social norm-constrained institutions, receive less coverage from analysts, and have higher expected returns (i.e., higher amounts of priced risk) than otherwise comparable stocks.

9 This is consistent with the framework of Bouslah et al. (2013) who argue that SP affects the firm's total risk through its impact on idiosyncratic risk because the implications of SP actions and practices are idiosyncratic to the firm. This is also in line with the literature relating firm idiosyncratic risk to corporate governance (e.g., Ferreira and Laux 2007).

10 For example, the GARCH model of Glosten et al. (1993) allows for an asymmetric effect on volatility of good and bad news.

11 It is important to note that KLD implemented several changes in its database during the sample period. For example, KLD added the Climate Change Concern in 1999 and the Management Systems strength in 2006 under the Environment dimension.

12 We also consider other measures of social performance (e.g., the sum of the differences between “total strengths” and “total concerns” of each KLD dimension for a given year, divided by the total number of KLD dimensions for that year). All these measures are highly positively correlated with the measures considered here and provide virtually the same results.

13 A similar approach is used by Deng et al. (2013) who find that aggregate social performance ("strengths" minus "concerns") has a significant positive effects on merger performance and the probability of its completion.

14
http://mba.tuck.dartmouth.edu/pages/faculty/ken.french/data_library.html.

15 Several studies use the market model to compute idiosyncratic risk (e.g., Malkiel and Xu 1997; Boutin-Dufresne and Savaria 2004; Lee and Faff 2009).

16 In addition to the above traditional risk measures which consider both upside potential and downside risk, we also compute lower partial moments (LPMs) as downside risk measures. LPMs consider only negative deviations of returns from a minimal acceptable return, e.g., zero, the risk-free rate or the average return (Homaifar and Graddy 1990; Sortino and Forsey 1996). Assuming that the target return is zero, the lower partial moment of order $n$ for firm $i$ is given by: ${\text{LPM}}_{i} \left( n \right) = \frac{1}{T}\mathop \sum \limits_{t = 1}^{T} { \hbox{max} }\left[ {\left( { - R_{it} } \right);0} \right]^{n}$ where $R_{it}$ is the daily return of firm $i$ at day $t$. LPM of order 0 (LPM0) can be interpreted as the shortfall probability (i.e., probability of loss when the target return is zero), whereas LPM of order 1 (LPM1) can be interpreted as the expected shortfall (or loss when the target return is zero). LPM of order 2 (LPM2) is the semi-variance around the target return. The higher the LPM order, the more risk averse the investor is (Sortino and Forsey 1996). We compute LPMs yearly for each firm-year observation using the previous year’s daily returns. The untabulated results for LPM1 through LPM3 are similar to those reported for total and idiosyncratic risks and are available from the authors upon request. It appears that the return distributions are reasonably symmetric so that all the correlations between total risk and LPM1 through LPM3 are 0.88 or higher. The mean (median) skewness of the daily returns is 1.29 (0.59) over the sample period (1991–2012). The mean (median) skewness of the daily returns is 0.64 (0.56) if we exclude the crisis period (2008 and 2009). In order to further examine the robustness of the main findings, we also calculated downside beta and LPM2 of the residuals using the four-factor model estimated with daily returns. The mean (median) downside systematic risk (downside market beta) is 1.165 (1.1). The mean (median) LPM2 of the residuals is 0.254 (0.225). LPM2 of the residuals is very highly correlated to total risk (0.92) and idiosyncratic risk (0.96). Similarly, the correlation coefficient between downside market beta and the market beta is high (0.77). The results for LPM2 of the residuals and downside systematic risk (downside market beta) are virtually similar to those reported in this paper. These untabulated results are available from the authors upon request.

17 The global financial crisis is commonly referred to in the literature as the “2008–2009 Global Financial Crisis.” Ait-Sahalia et al. (2012) use a Markov-Switching vector autoregression analysis of bond market data to identify the onset and end of the GFC (Global Financial Crisis) period as June 2007 and April 2009, respectively. Several papers select 2008 and 2009 as the years of the financial crisis including Murillo et al. (2011), Peters et al. (2012) and Lins et al. (2013).

18 Fama and French (1992, 1993); Berk et al. (1999); Carlson et al. (2004, 2006); Gebhardt et al. (2001); Botosan and Plumlee (2005); Gode and Mohanram (2003); Hail and Leuz (2006); Lee et al. (2009).

19 Gode and Mohanram (2003) argue that high B/M could reflect lower growth opportunities, lower accounting conservatism, or higher perceived risk.

20 Botosan and Plumlee (2005), Witmer and Zorn (2007) and Lee et al. (2009).

21 The firm’s expected return is expected to be positively related to stock return volatility and the firm’s beta (Gordon and Gordon 1997; Gode and Mohanram 2003; Botosan and Plumlee 2005; Hail and Leuz 2006; Lee et al. 2009) and to its idiosyncratic risk (Malkiel and Xu 1997; Lee and Faff 2009). However, the empirical evidence regarding the relation between market beta and expected return (proxied by the implied cost of equity) is mixed. For example, Gordon and Gordon (1997) and Gode and Mohanram (2003) find a positive relation, whereas Gebhardt et al. (2001) find no significant relation after controlling for the previous year’s average industry risk premium.

22 Brennan et al. (1998) find a negative relation between average returns and average dollar trading volume. Chordia et al. (2001) find a negative cross-sectional relation between average returns and both the level as well as the variability of liquidity, after controlling for size, book-to-market ratio, and momentum.

23 The *q*-theory of investment (e.g., Cochrane 1991; Liu et al. 2007) and the real options theory (e.g., Berk et al. 1999; Carlson et al. 2004, 2006) predict a negative relation between investment and risk. It follows that firms having higher investment-to-assets ratios should have lower risks than firms having lower investment-to-assets ratios.

24 Firm risk is expected to be negatively related to the size of its investor base (Merton 1987).

25 There are three potential sources of endogeneity: simultaneity bias; omitted variables bias; self-selection bias. Depending on the research question, one of two procedures is used to correct for endogeneity: Heckman two-step procedure for self-selection bias or Instrumental variable (IV) estimation.

26 The GMM estimation generates efficient estimates of the coefficients and consistent estimates of the standard errors that are robust to the presence of arbitrary heteroskedasticity and clustering by firm.

27 See Graves and Waddock (1994), Waddock and Graves (1997), McWilliams and Siegel (2000), Hillman and Keim (2001), Orlitzky and Benjamin (2001), Margolis and Walsh (2003), Orlitzky et al. (2003), Mattingly and Berman (2006), Barnea and Rubin (2010), Mahoney and Roberts (2007), Sharfman and Fernando (2008), and Harjoto and Jo (2011).

28 Harjoto and Jo (2011) show that analyst coverage is significantly related to SP. We do not include analyst coverage because it is highly correlated with firm size.

29 Except for the social performance measures and dummy variables, the variables are winsorized at the 1st and 99th percentiles to ensure that our results are not driven by outliers.

30 The mean (median) lower partial moment of order zero (LPM0) is 0.48 when the target return is zero. The distribution of LPM0 is reasonably symmetric (skewness is −0.25 and kurtosis is 3.11) indicating a 48 % probability of loss. This reinforces our observation noted earlier that the return distributions are reasonably symmetric so that all the correlations between total risk and LPM1 through LPM3 are very high. Our untabulated results for LPM1 through LPM3 are similar to those reported for total and idiosyncratic risks and are available from the authors upon request.

31 To determine the economic significance, we multiply the standard deviation of the SP score in the crisis period by the sum of the coefficients associated with SP and SP_crisis [i.e., 0.06 × (−0.004–0.138) = −0.00852 or 0.06 × (−0.02−0.201) = −0.01326]. Thus, an increase in SP by one standard deviation in the crisis period decreases the firm’s volatility by 0.852–1.326 % or about 1.84–1.18 % of that period’s average volatility [i.e., −0.00852/0.718 = −0.0118 or −0.01326/0.718 = 0.0184]. Disregarding the lack of statistical significance of the two corresponding coefficients for the pre-crisis period, the economic impact of a one standard deviation change in SP for the pre-crisis period is to decrease the firm's volatility by 0.028–0.14 % [i.e., 0.07 × −0.004 = −0.00028 or 0.07 × −0.02 = −0.0014].

32 Untabulated results show that the results for systematic risk are sensitive and depend on how beta is measured. For example, when using the IV regressions, the coefficient associated with SP becomes negative and significant when beta is measured using weekly returns. Also, the coefficient associated with Concerns (Con) becomes insignificant when beta is measured using weekly returns, and significantly positive when using downside beta instead of beta coupled with 3SLS as an estimation method.

33 The coefficient associated with Str_crisis is negative and significant only when the fixed effects method is used. In this case, the sum of the coefficients $(\alpha_{11} + \alpha_{12} )$ is negative and statistically significant suggesting a decrease of market beta of about 1.2 % during the financial crisis. However, this coefficient becomes insignificant when beta is measured using the CAPM model. Similarly, the sum of the coefficients $(\alpha_{11} + \alpha_{12} )$ becomes insignificant.

34 For example, Kappou and Oikonomou (2014) find that unethical transgressions are penalized more heavily than responsibility is rewarded. They find that the addition of a stock to a social index does not lead to material changes in its market price, whereas deletions are accompanied by negative cumulative abnormal returns.

35 The slack resources theory suggests that the availability of slack resources provides the opportunity for firms to improve their SP through CSR investments (McGuire et al. 1988; Waddock and Graves 1997).

36 We also computed two alternatives risk measures referred to as stock price crash risk (NSKEW and DUVOL) following Chen et al. (2001) and Kim et al. (2010). For a given year, NSKEW is the negative conditional skewness of firm-specific weekly returns. DUVOL is the natural logarithm of the ratio of the standard deviation of firm-specific weekly returns in the down weeks (i.e., below the weekly mean return over the previous year) to the standard deviation of firm-specific weekly returns in the up weeks (i.e., above the mean return). Firm-specific weekly return is defined as the natural logarithm of one plus the residual term from the CAPM model with lead and lag weekly market returns. None of the coefficients associated with the aggregate measure of SP or the two aggregate measures of strengths (Str) and concerns (Con) are significant when the dependent variable is the stock price crash risk measure. The only exception is obtained when using the IV estimation method where the coefficients associated with SP and Str (Con) are negative (positive) and significant. These untabulated results are available from the authors upon request.

37 The free cash flow to equity is computed as net income plus depreciation minus capital expenditures minus changes in non-cash working capital minus net debt issues minus preferred dividends. The free cash flow to the firm is computed as EBIT minus taxes paid plus depreciation minus capital expenditures minus changes in non-cash working capital.

38 The untabulated results are available from the authors upon request. The inclusion of some of these variables (e.g., bond rating or investment grade rating) significantly reduces the number of observations (not all firms are rated) and the goodness of fit of the model. The model used in this paper provides the highest R-square (i.e., tradeoff between model parsimony and the inclusion of additional explanatory variables).

39 The untabulated results of these additional tests are available from the authors upon request.

40 We thank an anonymous referee for pointing out this important issue.
